# Ultrastructural Synaptic Differences in the Central Inferior Colliculus in the 3xTG Mouse Across Three Disease Stages

**DOI:** 10.1002/cne.70120

**Published:** 2025-12-15

**Authors:** Jeffrey G. Mellott, Lena M. Dellaria, Madeline Guy, Sean R. Hergenrother, Nick J. Tokar, Dakota Z. Smallridge, Miljan Terzic, Jesse W. Young, Christine M. Dengler‐Crish

**Affiliations:** ^1^ Department of Biomedical Sciences Northeast Ohio Medical University Rootstown Ohio USA; ^2^ University Hospitals Hearing Research Center Northeast Ohio Medical University Rootstown Ohio USA; ^3^ Department of Pharmaceutical Sciences Northeast Ohio Medical University Rootstown Ohio USA

**Keywords:** 3xtg, aging, Alzheimer's disease, central inferior colliculus, synapse, ultrastructure

## Abstract

Numerous studies support a mechanistic link between hearing loss and increased risks of cognitive decline and dementia. Hearing loss is widely viewed as a modifiable risk factor for Alzheimer's disease (AD). During normal aging the inferior colliculus (IC), a large auditory midbrain nucleus, undergoes numerous changes to neurotransmission, and these changes contribute to the development of central gain and presbycusis. Recent reports also implicate the IC as a nucleus that undergoes processing changes during AD. We used transmission electron microscopy (EM) to examine the synaptic ultrastructure of the central IC (ICc) in 3xTG mice during presymptomatic, emerging, and established disease stages. Synapses were identified by a collection of presynaptic vesicles, a clear synaptic cleft, and a postsynaptic density. Symmetric synapses had pre and postsynaptic membranes of similar thickness, whereas asymmetric synapses had postsynaptic densities were conspicuously thicker than the presynaptic densities. We also quantified the presynaptic profile areas, active zone lengths, and presynaptic mitochondria. The data demonstrate a significant loss of symmetric and asymmetric synapses in the emerging disease stage. In particular, the density of symmetric synapses in the ICc was reduced by ∼50%. As inhibitory neurotransmitters gamma‐aminobutyric acid (GABA), glycine, and neuropeptide Y are released from neurons that form symmetric synapses in the IC, the robust loss of these synapses may contribute to central gain and presbycusis during AD. Furthermore, as these synapses were lost well before the established disease stages, perhaps alterations in ICc represent an early biomarker for Alzheimer's progression.

## Graphical abstract

Symmetric and asymmetric synapses were significantly downregulated by 50% and 20%, respectively, in the aging inferior colliculus of the Alzheimer's 3xTG mouse model during the emerging disease stage (7–8 months). There was no additional significant loss from the emerging disease stage into the established disease stage.

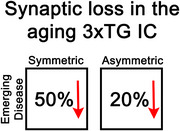

**Abbreviations**
AD: Alzheimer's disease
GABA: gamma‐aminobutyric acid
IC: inferior colliculus
ICc: central nucleus of the inferior colliculus
ICd: dorsal cortex of the inferior colliculus
IClc: lateral cortex of the inferior colliculus
LD: large dendrite
MD: medium dendrite
SD: small dendrite
Sp: spine

## 1 Introduction

Age‐related hearing loss (ARHL) and Alzheimer's disease (AD) are both chronic diseases that destroy the quality of life for many individuals and increase the healthcare burden for aging populations in nearly every society (Goman and Lin 2016; Helfer and Bartlett 2020; Gauthier et al. 2021). Although a relationship between hearing loss and dementia has been clinically observed for some time, a growing number of epidemiological studies and clinical investigations support the idea of possible mechanistic links between AD, ARHL, and cognitive decline (Ulhmann et al. 1989a; Uhlmann, Larson, et al. 1989; Lin et al. 2011, 2013; Deal et al. 2017; Heywood et al. 2017; Nadhimi and Llano 2021; Livingston et al. 2024; Chandra et al. 2025). Furthermore, hearing loss is now considered a modifiable risk factor for AD/dementia during middle age (Loughrey et al. 2018; Mukadam et al. 2020; Livingston et al. 2024; Chandra et al. 2025). Initial studies have found elevated levels of hallmark AD proteins amyloid beta and phosphorylated tau in brains of patients with ARHL (Zheng et al. 2022); and animal models of AD have demonstrated auditory deficits and neuroinflammation in auditory brain regions (Tsui et al. 2022; Paciello et al. 2021). Other studies have investigated explanations for AD and ARHL comorbidity that include communication deficits, depleted cognitive reserves, and cortical reorganization, but the reasons why heterogenous populations are often afflicted with both neurodegenerative conditions remain elusive (Griffiths et al. 2020; Nadhimi and Llano 2021; Llano et al. 2021; Chandra et al. 2025). Understanding the mechanistic foundations that link ARHL and AD may inform the development of therapeutic approaches designed to slow the progression of ARHL and AD.

Studies have suggested that processing deficits in the central auditory system precede the onset of AD (Gates et al. 2002). Inhibitory neurotransmission has been proposed as a link between central auditory dysfunction and AD, and increased hyperactivity (central gain) in the central auditory system may serve as an early biomarker to diagnose AD (Pérez‐González et al. 2022; Na et al. 2023). There is increasing evidence that brainstem structures are affected early in AD progression. Evidence of pathological change to these regions has been associated with a constellation of noncognitive signs and symptoms that may foreshadow future AD diagnosis (Rub et al. 2016; Tsui et al. 2022; Paciello et al. 2021). Of note, Na et al. (2023) found amyloid plaque accumulation in the inferior colliculus (IC) of 5XFAD Alzheimer's model mice at 6 months of age. The IC is a large auditory midbrain/brainstem nucleus comprised of three primary subdivisions and responsible for the integration and transformation of numerous ascending and descending sensory signals (see reviews; Cant 2005, Oliver 2005, Schofield 2005, Ito and Malmierca 2018). The central IC (ICc) is one of the three subdivisions and receives ascending lemniscal input that creates organized bands of synapses across a tonotopic axis (Oliver et al. 2005). The ICc contributes to central gain as inhibition is downregulated during normal aging, presumably as a compensatory mechanism to the lost peripheral excitation, and underlies a hyperactive environment during old age (Caspary et al. 2008; Auerbach et al. 2019, 2014; Parthasarathy, Bartlett, et al. 2019; Syka 2020; Ono and Ito 2024).

It is becoming increasingly appreciated that cochlear synapses are lost and hearing deficits occur, often undetected and untreated in many middle‐aged individuals (Parthasarathy et al. 2020; Wu et al. 2020, 2023; Guo et al. 2025; Zink et al. 2025). Middle age is also significant for when numerous changes occur to GABAergic neurotransmission in the IC (Milbrandt et al. 1997; Caspary et al. 1999; Robinson et al. 2019; Mafi et al. 2022; Wawrzyniak et al. 2025). Investigations of IC GABAergic synaptic ultrastructure in a normal aging model (the Fischer Brown Norway [FBN] rat) demonstrate several differences during middle and old age (Helfert et al. 1999; Mafi et al. 2022; Mellott et al. 2024; Wawrzyniak et al. 2025). Given that the IC in AD model mice accumulates amyloid beta plaques prematurely at what might be considered “middle” ages, this structure likely plays a role in central gain in models of AD and normal aging and undergoes changes to its synaptic ultrastructure between middle and old age during normal aging (Na et al. 2023). Here, we sought to determine if changes in synaptic ultrastructure in a widely characterized model of AD, 3xTG mice, resembled normal age‐related changes or whether the sequencing or nature of their synaptic changes exhibited a unique, pathological pattern.

In the current study, we used transmission electron microscopy (EM) to examine age‐related differences to the ultrastructure of symmetric (presumably inhibitory) and asymmetric (presumably excitatory) synapses in the lemniscal ICc of 3xTG mice at presymptomatic, emerging, and established disease stages of AD. The primary findings discovered from our analysis were (1) there was a significant loss of symmetric and asymmetric synapses between presymptomatic and emerging disease stages; however, there was not a further significant loss between the emerging and established groups. (2) Although the net loss of asymmetric synapses between “young” and “old” was similar to what has been reported in normal aging, the loss of symmetric synapses was substantially more robust (∼50%) and earlier in the 3xTG mice. There were additional ultrastructural changes that paralleled what had been reported in normal aging. Thus, it appears that inhibitory neurotransmission in the ICc may be significantly altered well before old age.

## 2 Materials and Methods

### 2.1 Animals

All procedures were conducted in accordance with the Northeast Ohio Medical University Institutional Animal Care and Use Committee and NIH guidelines. Results are described from 12 female 3xTG mice (B6;129‐Psen1^tm1Mpm^ Tg(APPSwe, tauP301L)1Lfa/Mmjax, RRID:MMRRC_034830‐JAX) developed on a hybrid B6129SF2/J background. Mice were stratified into three age groups based on previously published disease staging (Frame et al. 2022; Paciello et al. 2021) as follows: presymptomatic (2 months) where there is little to no amyloid beta (Aß) accumulation in areas of primary brain expression (hippocampus and entorhinal cortex); emerging disease (8 months) where there is increased accumulation of Aß in brain, neuroinflammation, and emerging cognitive deficits; established disease (15–19 months) characterized by advanced age and has significant accumulation of Aß, neurofibrillary tau, neuroinflammation, and pronounced cognitive deficits. The 3xtg‐AD mouse model has been widely used and its progression of amyloid and tau pathology is well‐documented across numerous studies, making its pathological staging highly reliable and broadly accepted in the field (Oddo et al. 2003). Efforts were made to minimize the number of animals and their suffering.

### 2.2 Perfusion and Sectioning

Animals were deeply anesthetized with pentobarbital and perfused transcardially with Tyrode's solution, followed by 200 mL of 1% glutaraldehyde and 4% paraformaldehyde in 0.1 M phosphate buffer at a pH 7.4. We chose to use a lower glutaraldehyde concentration (concentration for non‐immuno EM studies is often 2%–2.5%) as we plan to investigate the expression of various peptides (that require a weaker fixative to achieve successful immunogold labeling). Given that we achieved quality ultrastructure to analyze, this will allow us to explore a wide range of immunomarkers in the ultrastructure of the 3xTG mouse without the need for additional aged populations. After the brain was removed, it was stored at 4°C in the same fixative it was perfused with in 0.1 M phosphate buffer. The brain was prepared the next morning by blocking the brain with transverse cuts posterior to the cochlear nucleus and anterior to the thalamus. The tissue was then cut into 50 µm thick transverse sections with a Vibratome (VT1000S, Leica Microsystems, Buffalo Grove, IL, USA). The tissue was collected in four series. Series were processed as described below or stored in freezing buffer for future processing.

### 2.3 Tissue Processing for EM

**FIGURE 1:**
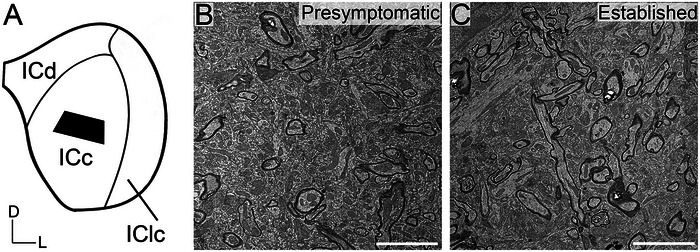
(A) Schematic illustrating approximating where tissue (black trapezoid) was extracted from the ICc in each case. (B) A montage, 20 µm by 20 µm, of ICc tissue from a presymptomatic disease stage 3xTG mouse. (C) A montage, 20 µm by 20 µm, of ICc tissue from an established disease stage 3xTG mouse. Scale bars = 5 µm. D, dorsal; ICc, central inferior colliculus; ICd, dorsal cortex of the IC; IClc layer I, the first layer of the lateral cortex of the IC; IClc layer II, the second layer of the IClc; IClc layer III, the third layer of the IClc; L, lateral.

Besides the fixative concentration, tissue was processed similar to our previous EM studies (Mafi et al. 2022; Mellott et al. 2024; Wawrzyniak et al. 2025). Briefly, tissue was postfixed in 1% osmium tetroxide for 30 min, dehydrated in a series of alcohols (50%, 70%, 95%, 100%, and 2x propylene oxide), embedded in Durcupan resin (Sigma‐Aldrich; Millipore Sigma, Burlington, MA, USA), and flat‐mounted between sheets of Aclar Embedding Film (Ted Pella Inc., Redding, CA, USA) at 60°C for 48–72 h. Mid‐rostrocaudal IC sections (between interaural levels −1.40 and −1.28 mm; Paxinos and Franklin 2019) were examined with brightfield stereomicroscopy. Trapezoidal blocks, with a 0.5 mm base and 0.3–0.4 mm height, were extracted from the middle of the ICc (Figure 1). One “block” of tissue was taken from each animal processed in the study. Initial borders of the ICc were delineated according to the mouse anatomical atlas of the brain (Paxinos and Franklin 2019). Osmium fixation revealing the conspicuous lateral lemniscal fibers, libraries of decarboxylase (GAD) immunoreactivity in EM‐prepared tissue, and adjacent sections reacted for Nissl, and our experience with EM in the IC further guided our block trimming to best ensure tissue was from ICc (Nakamoto et al. 2013; Mellott et al. 2014; Mafi et al. 2022; Mellott et al. 2024; Wawrzyniak et al. 2025). Tissue blocks were glued to a cylindric resin base with cyanoacrylate (Krazy Glue, Columbus, OH, USA). IC sections with removed tissue blocks were then imaged for record keeping and representative comparison between cases.

Ultrathin sections (50 nm) were taken with an ultramicrotome (UC6 Ultramicrotome, Leica Microsystems, Buffalo Grove, IL, USA). For each tissue block, every twentieth section was collected onto a 300‐mesh Formvar‐coated nickel mesh grid (Electron Microscopy Science, Hatfield, PA, USA) to better ensure a singular synapse was not analyzed twice. A total of eight grids, each with a single ICc ultrathin section.

It is broadly accepted that terminals forming symmetric (type II) synapses in the IC are inhibitory and release gamma‐aminobutyric acid (GABA) or glycine (Shneiderman and Oliver 1989; Paloff and Usunoff 1992; Helfert et al. 1999; Liu et al. 1999; Nakamoto et al. 2013, 2014). To confirm this relationship in the 3xTG mouse IC, we ran post‐embedding immunogold for GABA on a second series of tissue from one case in each disease group. Briefly, (see: Nakamoto et al. 2013; Mellott et al. 2014; Mafi et al. 2022; Mellott et al. 2024; Wawrzyniak et al. 2025), once sections were placed on grids, they were dried for 3 h and were then placed overnight into anti‐GABA antibody (rabbit anti‐GABA, Sigma, St. Louis, MO) diluted 1:500 in 0.05 M Tris‐buffered saline with 0.1% Triton X‐100, pH 7.6 (TBST). The next day the sections are washed in TBST pH 7.6, then washed in TBST pH 8.2, and placed into a secondary antibody conjugated to 15 nm gold particles (goat anti‐rabbit, diluted 1:25 in TBST pH 8.2; Ted Pella Inc., Redding, CA). All sections, post‐embedded or not, were washed in TBST pH 7.6, washed in Nanopure water, stained with uranyl acetate (2% aqueous) and Reynold's lead citrate (Reynolds 1963) to provide contrast, and air‐dried.

### 2.4 EM Imaging

Twelve blocks of tissue from 12 female 3xTG mice with superior ultrastructure were chosen. A 5‐point scale was used to grade the intactness and quality of ultrastructure. Only tissue with a score of 4 or 5 was quantified. Our 5‐point scale reflects a combination of successful fixation and absence of electron dense artifacts. Scores of 4 and 5 yield clear ultrastructure with easily identifiable symmetric and asymmetric synapses that are readily resolved. Tissue scored as a 3 yields ultrastructure that can be qualitatively analyzed; however, membrane intactness is not preserved such that quantitative data can be consistently obtained. Tissue scored as a 1 or 2 has severe defects in the pre and postsynaptic membranes resulting in uninterpretable synaptic profiles and was not used. Ultrastructure of the ICc was imaged with a transmission electron microscope (JEM‐1400Plus, JEOL, Peabody, MA, USA) at an accelerating voltage of 80 kV and at a magnification of 50,000. On the basis of experience, a magnification of 50,000 ensures that symmetric synapses in the IC are discernable. Tissue was digitally imaged and rendered with a Rio9 side mount camera (Gatan, Pleasanton, CA, USA). Images of ultrastructure were taken with Gatan Microscopy Suite Software (GMS3, Gatan, Pleasanton, CA, USA) integrated and calibrated with SerialEM Tomography software (Mastronarde 2003). SerialEM is a gold standard for analytical applications in biological TEM and facilitated efficient imaging, analysis, and data recording. For each tissue block, we collected 400 µm^2^ (22,214 × 22,214 pixels; Figure 1B,C) montages across eight grids for a total of 3200 µm^2^. Adobe Photoshop (Adobe Systems Inc., San Jose, CA, USA) was used to add scale bars, crop images, adjust intensity levels, and colorize monochrome images.

### 2.5 Analysis of Symmetric and Asymmetric Synapses

**FIGURE 2:**
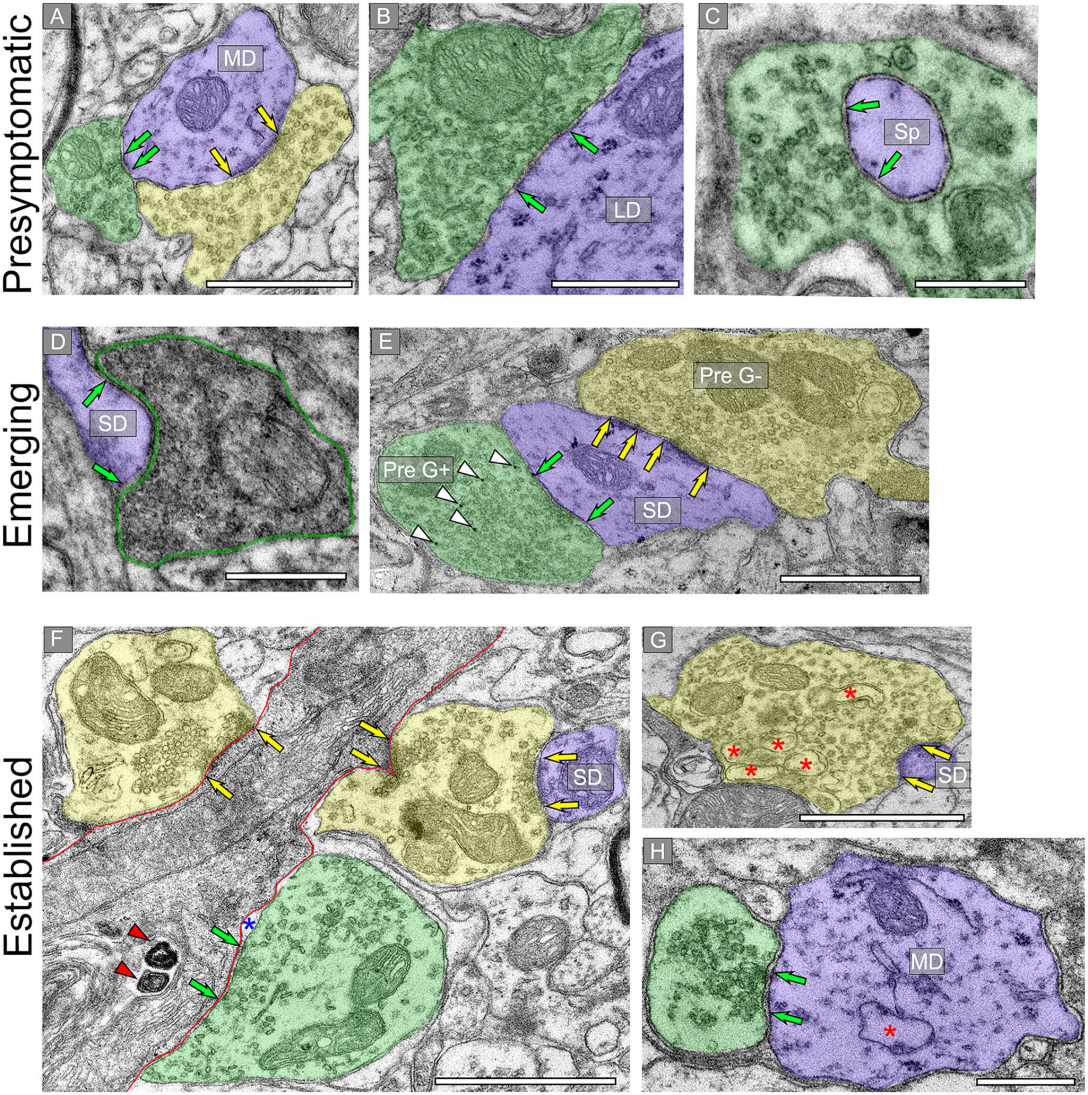
Electron micrographs across three disease stages showing symmetric (green arrow pairs) and asymmetric (yellow arrow pairs) synaptic ultrastructure in the central inferior colliculus (ICc). Presynaptic profiles forming symmetric synapses are pseudocolored green. Presynaptic terminals forming asymmetric synapses are pseudocolored yellow. Postsynaptic structures are pseudocolored blue. (A–C) Electron micrographs showing examples of symmetric and asymmetric synapses on dendrites and spines the ICc of presymptomatic disease stage tissue. (D–F) Electron micrographs showing examples of symmetric and asymmetric synapses on dendrites in the ICc of emerging disease stage tissue. (D) A darkened presynaptic terminal forming a symmetric synapse. (E) A small dendrite receiving both a symmetric and asymmetric synapse. Tissue was postembedded for immunogold (white arrowheads) for GABA to demonstrate that GABAergic presynaptic terminals (Pre G+) form symmetric synapses, whereas GABAnegative presynaptic terminals (Pre G−) often formed asymmetric synapses. (G–I) Electron micrographs showing examples of symmetric and asymmetric synapses in the ICc of established disease stage tissue. (F) A darkened dendrite (red outline) targeted by both symmetric and asymmetric synapses. Dark whorls/membrane inclusions can be seen (red arrowheads). Gaping can be seen near the symmetric synapse (blue asterisk). (G) A presynaptic terminal forming an asymmetric synapse with a small dendrite. The terminal has numerous occurrences of membrane debris (red asterisk). (H) Presynaptic terminal forming a symmetric synapse with a medium dendrite that has membrane debris (red asterisk). Presymptomatic—2 months; emerging—8 months; established—15–19 months. Scale bars = 500 nm. LD, large dendrite; MD, medium dendrite; P SD, small dendrite; Sp, spine.

Synapses were identified by a collection of presynaptic vesicles, a clear synaptic cleft, and a postsynaptic density (PSD). Synapses were classified as symmetric [pre and postsynaptic membranes were of similar thickness] or asymmetric [postsynaptic densities were conspicuously thicker than the presynaptic densities] (Rockel and Jones 1973; Paloff and Usunoff 1992; Helfert et al. 1999; Nakamoto et al. 2013; Mafi et al. 2022; Mellott et al. 2024). As terminals that form symmetric synapses are likely positive for GABA or glycine, the presynaptic vesicles were often pleomorphic; whereas terminals forming asymmetric synapses regularly had vesicles that were rounder, slightly larger, and more uniform in shape (Figure 2).

### 2.6 Data Analysis

We examined 12,800 µm^2^ of ICc tissue across four presymptomatic disease stage tissue blocks, 12,800 µm^2^ of ICc across four emerging disease stage tissue blocks, and 12,800 µm^2^ of ICc tissue across four established disease stage tissue blocks (Tables 1 and 2). Each presynaptic terminal and postsynaptic target was analyzed manually with ImageJ (Schneider et al. 2012). We collected several ultrastructural details for each synapse: (1) presynaptic area (we assigned presynaptic areas of >1.0 µm^2^ as “large” and those with areas <0.6 µm^2^ as “small”), (2) active zone length, (3) the number of total vesicles in the terminal and the number of those vesicles at the active zone (releasing pool), and (4) the number and scored the ultrastructure of each presynaptic mitochondria (Smith et al. 2016; Mafi et al. 2022; Wawrzyniak et al. 2025). We recorded the postsynaptic target of each synapse as either a soma, large dendrite (LD), medium dendrite (MD), small dendrite (SD), or spine (Sp). For the sake of consistency across EM studies on the aging IC, we used dendritic sizes (>1.5 µm‐large; 0.5–1.5 µm‐medium; <0.5 µm‐small; Helfert et al. 1999; Mafi et al. 2022; Mellott et al. 2024; Wawrzyniak et al. 2025). As detailed in Mafi et al. (2022), it is sometimes difficult to distinguish Sps from SDs in the IC, as (1) there is overlap in diameter ranges, (2) Sp necks are not consistently observed at 50 nm thick sections, and (3) Sp apparatus is only present in a subset of Sps.

**TABLE 1:** Summary of the ultrastructural characteristics of symmetric synapses in the central nucleus of the inferior colliculus (ICc).

Presymptomatic (months)	Area examined (µm^2^)	Symmetric synapses	Bouton area (µm^2^)	Synaptic length (nm)	Ave. # mito	Mito grade	Vesicles (at synapse)	Vesicles (total)
B217—2	3200	245	0.62	265.2	0.76	4.6	3.4	44.1
B218—2	3200	247	0.6	303.6	0.71	4.6	6.2	73.3
B219—2	3200	189	0.8	308.6	1.3	4.5	4.4	56.4
B232—2	3200	209	0.5	267.5	0.62	4.4	6.4	65.7
Totals/Averages	**12,800**	**890**	**0.63**	**285.5**	**0.82**	**4.6**	**5.1**	**60.3**
**Emerging**								
B255—7.5	3200	105	0.67	239	1.3	4.2	3.3	32.6
B256—7.5	3200	124	0.68	249.6	1.3	4.1	3.1	37.8
B257—7.5	3200	69	0.57	246.1	1.2	4	7.2	27.7
B259—7.5	3200	112	0.62	297.9	1.3	4.1	5.9	67.4
Totals/Averages	**12,800**	410**	**0.64**	**260.1**	**1.3**	4.1**	**4.7**	**43.2**
**Established**								
B210—15	3200	103	0.88	237.6	1.3	3.6	2.2	43.1
B211—19	3200	130	0.8	299.3	1.1	3.8	5.1	67.2
B212—19	3200	66	0.64	241	0.66	3.9	2.8	37.2
B213—19	3200	91	0.89	266.6	1.6	3.7	2.6	38.3
Totals/Averages	**12,800**	390**	0.84*	**265.5**	**1.2**	3.7**^	3.4*^	**49**

*p < 0.05 as compared to presymptomatic.

**p < 0.01 as compared to presymptomatic.

^*p* < 0.05 as compared to emerging.

#*p* < 0.05 as compared to established.

Variation in synaptic density, presynaptic area, the active zone size, number of vesicles, presynaptic mitochondria, and postsynaptic targets according to age group and frequency region were analyzed using linear mixed‐effects models. Mixed‐effects models allow for a hybrid of repeated measures analysis (i.e., “within‐subject” variables), Model I ANOVA fixed factor analysis (i.e., “between‐subject” variables), and Model II ANOVA random factor analysis (i.e., variance components), in the same gestalt statistical test. In this study, disease stage was specified as a between‐subjects fixed factor across individual rats, ultrastructural profile (symmetric or asymmetric) was specified as a within‐subjects fixed factor within individual rats, and individual rat number was specified as a random factor. Therefore, individual animal was the unit of analysis used to set degrees of freedom for age group comparisons, whereas individual synapses within animals were the unit of analysis used to set degrees of freedom for comparisons between symmetric and asymmetric synapses. Several types of mixed‐effects models were used to analyze the data, depending on the nature of the response (dependent) variable. We used standard linear mixed‐effects models to analyze variation in continuous variables (i.e., terminal size and active zone length). Both variables were log‐transformed prior to model fitting to better approximate normality of residuals. To analyze variation in most count variables (i.e., number of synapses, number of vesicles present at the active zone, and number of mitochondria), we used generalized linear mixed‐effects models, specifying a Poisson distribution for the error term. In contrast, we found that a mixed‐effects linear model of (log‐transformed) total vesicle number was a better fit to the data than a generalized linear model and therefore elected to use the linear model in this case. Finally, we used a mixed‐effects cumulative link model to examine variation in ordinal‐scale mitochondrial grades. *p* values for pairwise post hoc tests of differences between disease stage groups were adjusted using the False Discovery Rate procedure (Benjamini and Hochberg 1995), a method that simultaneously limits experiment‐wise alpha inflation and minimizes the correlated loss of statistical power. To mitigate the impact of heteroscedasticity among groups, degrees of freedom for post hoc comparisons were adjusted using the Kenwood‐Roger method (Kenward and Roger 1997).

To assess dispersion among rates within age groups, we calculated the intra‐class correlation coefficient (ICC) for each our mixed‐effects models. The ICC quantifies proportion of total variance accounted for by interindividual differences (Nakagawa et al. 2017). ICCs for our models ranged from 0.003 to 0.22, signaling that 78%–99.7% of the total variance in our dependent measures was independent of interindividual differences among rats. We are therefore confident that the results discussed below are not due the influence of a subset of animals.

All statistical tests were performed in R (version 4.5.2 for Mac OS X; R Core Team 2025), supplemented by the add‐on packages *emmeans* (Lenth 2019), *lme4* (Bates et al. 2015), *lmerTest* (Kuznetsova et al. 2017), *ordinal* (Christensen 2023), *performance* (Lüdecke et al. 2021), and *tidyverse* (Wickham et al. 2019).

## 3 Results

We examined the ultrastructure of synapses forming symmetric and asymmetric synapses in three disease stage groups (presymptomatic [2 months]; emerging [8 months]; established [15–19]) in the ICc of female 3xTG mice. We quantified 1690 symmetric and 1814 asymmetric synapses across 38,400 µm^2^ of tissue (Tables 1 and 2). Our primary finding was that the density of symmetric and asymmetric synapses declined significantly beginning at emerging disease stages (Tables 1 and 2). We found significant differences within terminal sizes and mitochondrial ultrastructure; however, our analyses did not detect significant changes across active zone lengths and vesicles pools. We first present the characterization of synaptic types along with their decline in synaptic density. We then present data regarding the significant changes to terminal size and mitochondria grade. Lastly, we describe the frequency of which symmetric and asymmetric synapses form onto somata, dendrites, and Sps.

**TABLE 2:** Summary of the ultrastructural characteristics of asymmetric synapses in the central nucleus of the inferior colliculus (ICc).

Presymptomatic (months)	Area examined (µm^2^)	Asymmetric synapses	Bouton area (µm^2^)	Synaptic length (nm)	Ave. # mito	Mito grade	Vesicles (at synapse)	Vesicles (total)
B217—2	3200	197	0.59	263.4	0.92	4.5	4.5	52.8
B218—2	3200	164	0.6	295.5	0.61	4.6	6.3	78.3
B219—2	3200	195	0.5	337.6	0.59	4.5	6.6	68.1
B232—2	3200	175	0.5	269.6	0.57	4.5	6.9	69.6
Totals/Averages	**12,800**	**731**	**0.55**	**292.2**	**0.68**	**4.5**	**6.1**	**66.9**
**Emerging**								
B255—7.5	3200	152	0.71	255.8	1.4	4.4	4.4	37.1
B256—7.5	3200	124	0.79	264.8	1.4	4.1	4.1	45.4
B257—7.5	3200	118	0.57	244.1	1	4.2	7.8	30.9
B259—7.5	3200	189	0.5	274.7	0.7	4.2	5.6	64.9
Totals/Averages	**12,800**	583*	**0.63**	**261.6**	**1.1**	4.2**	**5.4**	**46.4**
**Established**								
B210—15	3200	98	0.79	244	1	4.1	2.6	39.8
B211—19	3200	119	0.8	297.2	1.1	3.9	5	63.1
B212—19	3200	130	0.62	253	0.59	4	3.1	40.3
B213—19	3200	153	0.9	306.9	1.8	3.9	3.1	44.2
Totals/Averages	**12,800**	500**	0.8*	**278.1**	**1.2**	4.0**^	3.4**^	**46.9**

*p < 0.05 as compared to presymptomatic.

**< 0.01 as compared to presymptomatic.

^*p* < 0.05 as compared to emerging.

#*p* < 0.05 as compared to established.

### 3.1 Characterization of Synapses and Synaptic Loss

Symmetric and asymmetric synapses were readily found throughout the IC tissue of mice from each disease stage. (Figure 2, arrow pairs [green‐symmetric; yellow‐asymmetric]). Most symmetric and asymmetric synapses were found contacting dendrites of various sizes (Figure 2A,B,D–H). When a dendritic Sp was the postsynaptic target, it often appeared to be engulfed by the terminal (Figure 2C). Terminals forming symmetric synapses are often inhibitory; an example of this is shown in Figure 2E where post‐embedding immunogold labeling for GABA (white arrowheads) is shown in tissue from an emerging disease stage case. We observed more frequent evidence of synaptic abnormalities in tissue from 3xTG mice at emerging (Figure 2D,E) and established disease stages (Figure 2F–H). Figure 2D shows a darkened terminal—often an indication of degeneration and injury—at a symmetric synapse (green arrows) on a SD in tissue from an emerging disease stage mouse. Figure 2F demonstrates several synaptic abnormalities in IC tissue from an established disease stage 3xTG mouse, including a darkened dendrite receiving two asymmetric (yellow arrow pairs) synapses and one symmetric (green arrow pair) synapse, membrane whorls (red arrowheads), and swelling/gaping adjacent to an active zone (blue asterisk). We also commonly observed irregular inclusions of membrane in tissue from established disease stage 3xTG mice (Figure 2G,H; red asterisks).

**FIGURE 3:**
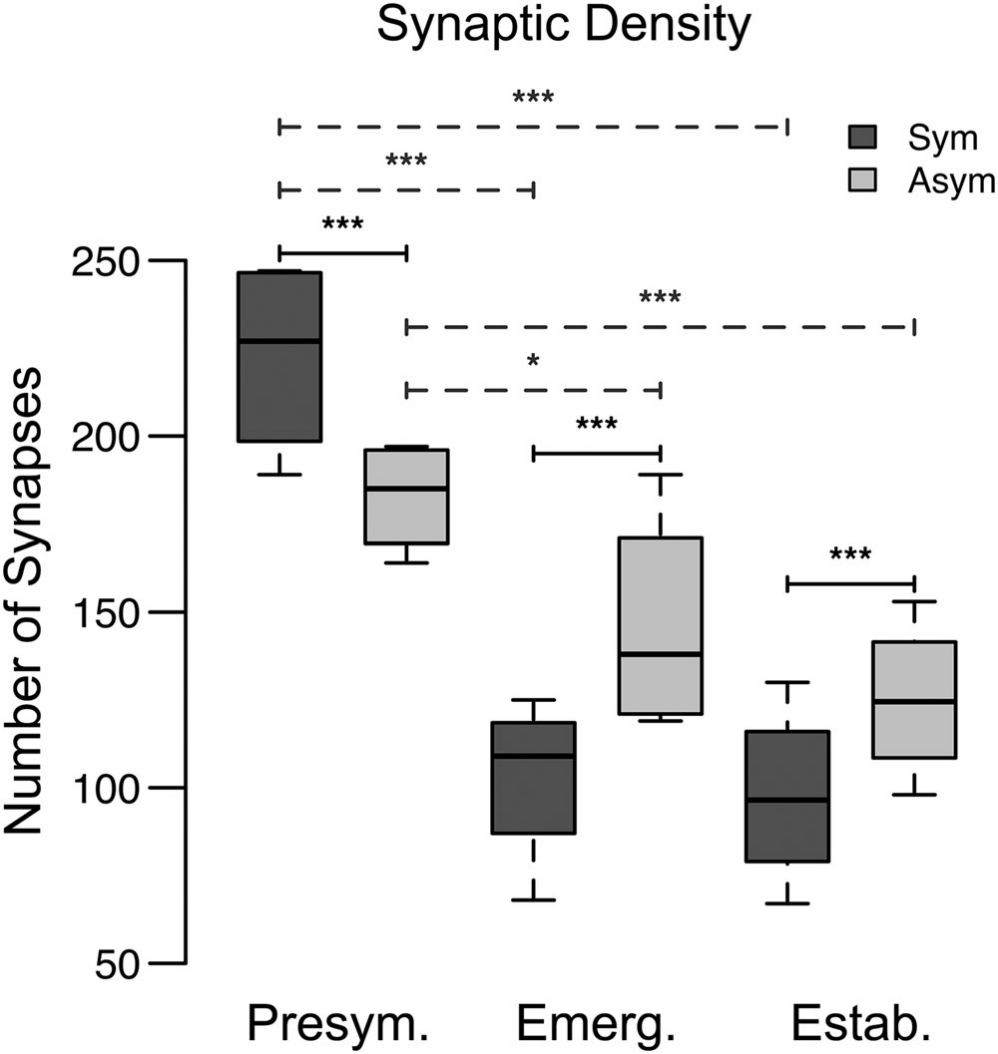
Synaptic density declines across disease stages. Box plots summarizing the decrease in synaptic density of symmetric and asymmetric synapses in the ICc of presymptomatic, emerging, and established disease stage tissue. Pairwise differences demonstrated a significant decrease in the density of symmetric synapses between presymptomatic and emerging stages (***p < 0.0001) and between presymptomatic and established stages (***p < 0.0001). There was no difference between emerging and established stages (*p* = 0.88). Pairwise differences demonstrated a significant decrease in the density of asymmetric synapses between presymptomatic and emerging stages (*p < 0.02) and between presymptomatic and established stages (***p < 0.0001). There was no difference between emerging and established stages (*p* = 0.24). Pairwise differences demonstrated that the density of symmetric synapses was greater than the density of asymmetric synapses in presymptomatic tissue (***p = 0.001). At emerging and established stages, pairwise differences demonstrated that asymmetric synapses had a higher density than symmetric synapses (***p < 0.0001; ***p = 0.0002). In each box plot, dark lines represent the median of the distribution, boxes extend across the interquartile range, and whiskers extend to ±150% of the interquartile range. Circles indicate outliers beyond this range. Presymptomatic—2 months; emerging—8 months; established—15–19 months. Each age group had four cases.

At presymptomatic ages, the number of symmetric synapses was significantly greater than the number of asymmetric synapses in 3xTG IC tissue (Figure 3); however, in emerging and established disease stage tissue, we found the opposite effect. Although both types of synapses were reduced in these two disease stage groups, symmetrical synapses were most severely downregulated, resulting in their significantly lower abundance than asymmetrical synapses in both age groups (Figure 3; Tables 1 and 2).

#### 3.1.1 Symmetric Synaptic Loss

We quantified 890 symmetric synapses across 12,800 µm^2^ of IC tissue (Figure 3, Table 1) from presymptomatic disease stage 3xTG mice. In contrast, we counted only 410 symmetric synapses in the same total tissue area from emerging disease stage mice, representing a significant decrease between these two age groups (*p* < 0.0001; Figure 3, Table 1). Symmetric synapse counts (*n* = 390) in tissue from established disease stage were also reduced relative to counts obtained from presymptomatic stage mice (*p* < 0.0001) but did not differ from counts in the emerging disease stage group (*p* = 0.63; Figure 3, Table 1).

#### 3.1.2 Asymmetric Synaptic Loss

We quantified 731 asymmetric synapses across 12,800 µm^2^ of IC tissue (Figure 3, Table 2) from presymptomatic disease stage 3xTG mice; asymmetric synapse counts were significantly reduced to 583 in emerging disease stage mice (*p* = 0.0284; Figure 3, Table 2) and to 500 in established disease stage mice (*p* = 0.0001). Asymmetric synapse counts did not differ between emerging and established disease stage IC (*p* = 0.1064; Figure 3, Table 1).

### 3.2 Terminal Size

**FIGURE 4:**
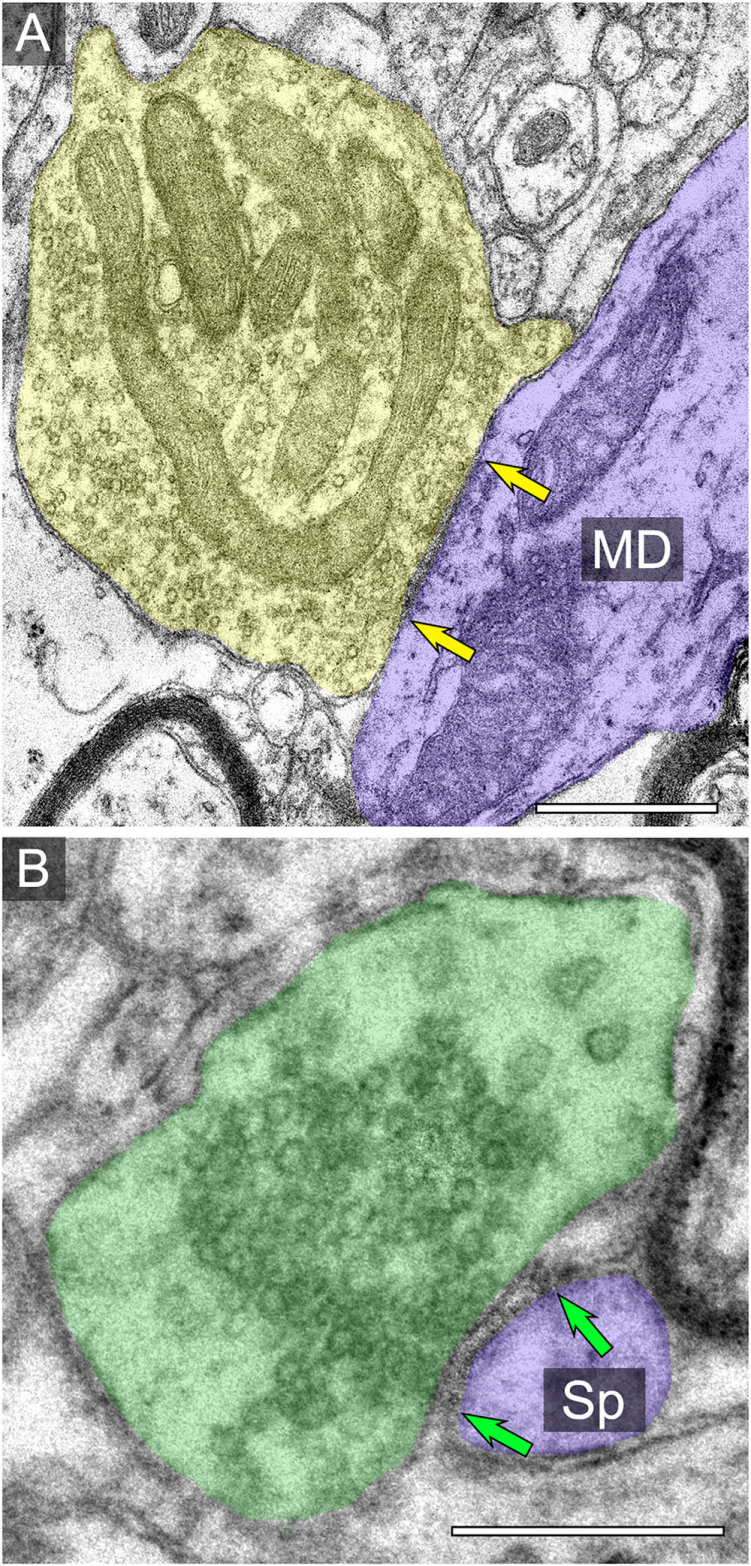
Electron micrographs of two synapses at an established disease stage showing an asymmetric (yellow arrow pair) and a symmetric (green arrow pair) synaptic ultrastructure in the ICc. Presynaptic terminal forming an asymmetric synapse is pseudocolored yellow. Presynaptic profile forming a symmetric synapse is pseudocolored green. Postsynaptic structures are pseudocolored blue. (A) Electron micrograph of a large (>1.0 µm^2^) presynaptic terminal forming an asymmetric synapse on a medium dendrite. (B) Electron micrograph of a large (>1.0 µm^2^) presynaptic terminal forming a symmetric synapse on spine. Scale bar = 500 nm. MD, medium dendrite; Sp, spine.

**FIGURE 5:**
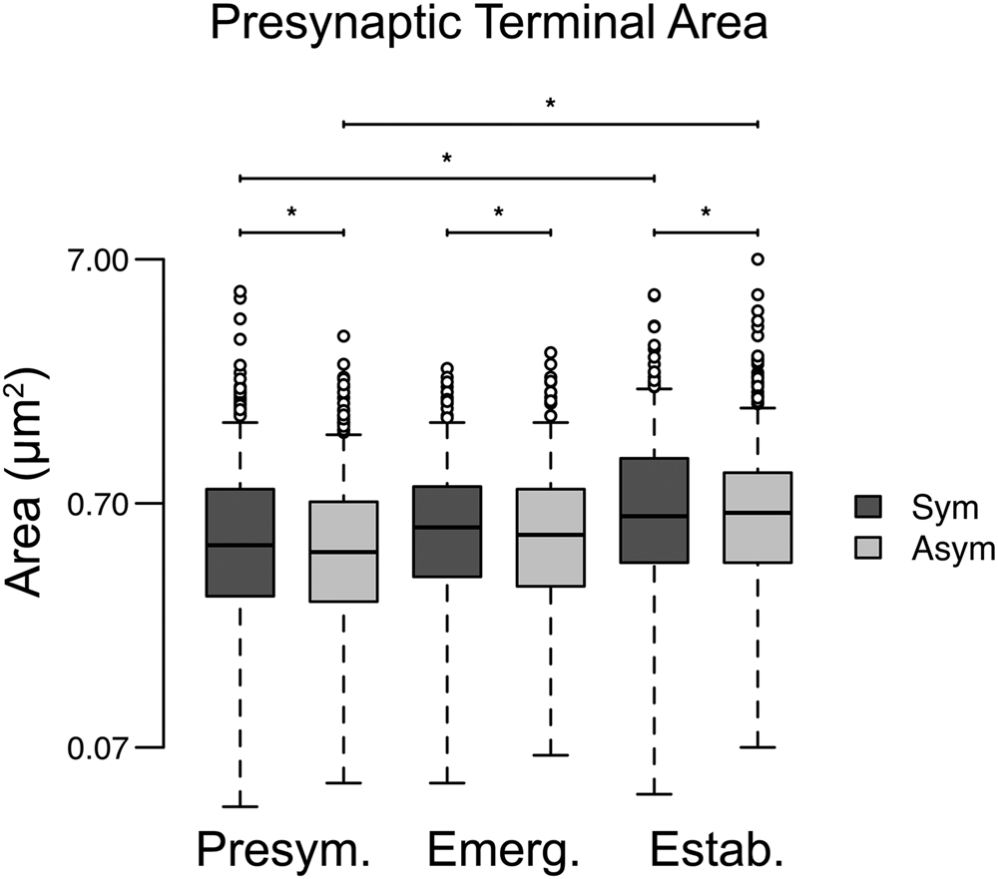
The average presynaptic terminal area increased at an established disease stage. Box plots summarizing the increase in presynaptic terminal area in the ICc between disease stages. Pairwise differences demonstrated a significant increase in presynaptic terminal area between presymptomatic and established stages regardless of synaptic type (*p < 0.019). Pairwise differences were not demonstrated, regardless of synaptic type, between presymptomatic and emerging stages (*p* = 0.2) or between emerging and established stages (*p* = 0.09). There was no difference between emerging and established stages (*p* = 0.88). At each disease stage, pairwise differences demonstrated that the average presynaptic terminal that forms a symmetric synapse is larger than the average presynaptic terminal that forms an asymmetric synapse (*p* = 0.012). In each box plot, dark lines represent the median of the distribution, boxes extend across the interquartile range, and whiskers extend to ±150% of the interquartile range. Circles indicate outliers beyond this range. Presymptomatic—2 months; emerging—8 months; established—15–19 months. Each age group had four cases.

We examined the area of each presynaptic terminal forming a synapse at each disease stage. It was more common to observe larger (>1.0 µm^2^) presynaptic terminals forming either symmetric or asymmetric synapses in the established disease stage (Figure 4), and this was supported by the finding that average area of presymptomatic terminals forming symmetric or asymmetric synapses was significantly larger in the established disease stage as compared to the presymptomatic (*p* = 0.0188; Figure 5; Tables 1 and 2). There were no differences in terminal size between emerging and presymptomatic stages (*p* = 0.1966) or between established and emerging stages (*p* = 0.0906; Figure 5; Tables 1 and 2). Regardless of disease stage, presynaptic terminals forming symmetric synapses were significantly larger (*p* = 0.0117) than presynaptic terminals forming asymmetric synapses (Figure 5; Tables 1 and 2).

### 3.3 Vesicle Pools and Active Zone Lengths

The number of vesicles present at the active zone for presynaptic terminals forming symmetric synapses was significantly decreased at the established (*p* < 0.05) disease stage (Table 1). Vesicles at the active zone were also reduced in the presynaptic terminals forming asymmetric synapses in established (*p* < 0.01) disease stage (Table 2).

The average length of each active zone (synaptic length) and the total number of vesicles in each presynaptic terminal for symmetrical and asymmetrical synapses are provided in Tables 1 and 2, respectively. No significant differences in these data sets were indicated between any age groups for either symmetrical (Table 1) or asymmetrical (Table 2) synapses.

### 3.4 Mitochondria Ultrastructure

**FIGURE 6:**
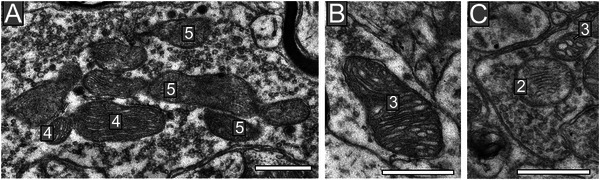
Ultrastructural health grades of mitochondria in presynaptic mitochondria. Electron micrographs demonstrating mitochondria that were graded on a scale of 1–5 (1‐worst; 5‐best) based on the condition of the cristae's ultrastructure. Mitochondria with numerous, intact, and non‐swollen cristae and electron dense cytoplasm were graded 5. Mitochondria with severely fragmented or missing cristae were graded as 1 or 2. (A) A presynaptic terminal with many mitochondria that were scored a 5 or 4. (B) A mitochondria scored a 3. However, this is a good example of the subjectiveness of the approach as this is close to being scored a 4. (C) Relative examples of a mitochondria scored a 3 as the cristae are swollen, and a pale mitochondrion (2) as the cytoplasm is no longer electron dense. In general, scores of 2 and 1 were rare.

**FIGURE 7:**
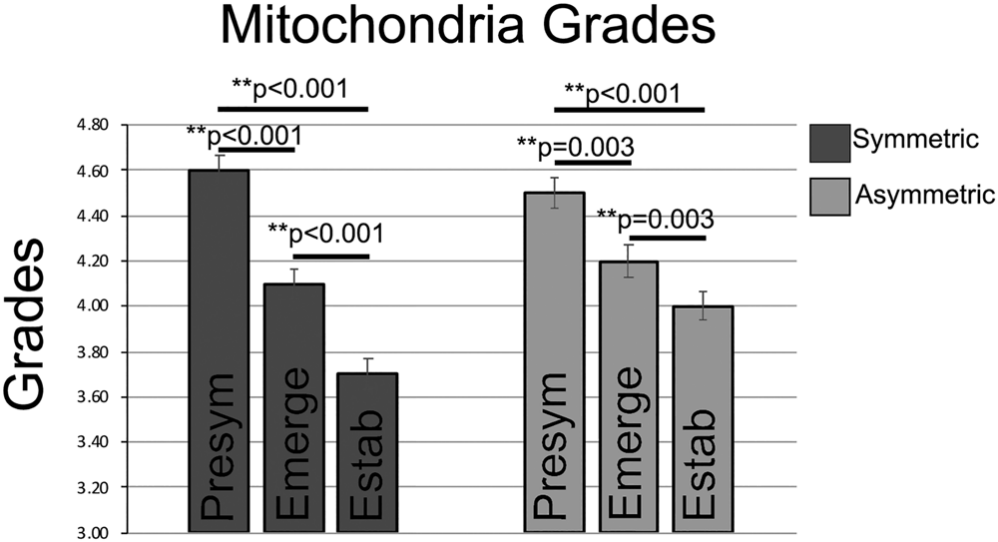
Mitochondria ultrastructural scores decline between disease stages in the ICc. Bar graphs summarizing the mitochondrial scores in the presymptomatic, emerging, and established disease stage ICc tissue. Pairwise differences demonstrated a significant decrease of scores in presynaptic terminals forming symmetric synapses between presymptomatic and emerging disease stages (**p < 0.001); between presymptomatic and established disease stages (**p < 0.001); between emerging and established disease stages (**p < 0.001). Pairwise differences demonstrated a significant decrease of scores in presynaptic terminals forming asymmetric synapses between presymptomatic and emerging disease stages (**p < 0.001); between presymptomatic and established disease stages (**p = 0.003); between emerging and established disease stages (**p = 0.003). Presymptomatic—2 months; emerging—8 months; established—15–19 months. Each age group had four cases.

The ultrastructure of each presynaptic mitochondria was scored to reflect the intactness of its membranes and cristae and electron density of its cytoplasm using the following grading scale: 1 indicating poor quality/lack of intactness; 5 representing excellent quality/complete intactness (Figure 6). The average mitochondria grade for symmetric synapses (Table 1) significantly decreased with increasing disease stage in 3xTG mice (Figure 7). Mitochondria grades for symmetric synapses in both emerging (*p* < 0.0001) and established (*p* < 0.0001) disease stages were significantly reduced relative to presymptomatic stage scores with established disease stage mitochondria exhibiting significantly lower scores than emerging disease stage mitochondria (*p* < 0.0001; Figure 7).

For asymmetric synapses (Table 2), mitochondrial grades also decreased in a stepwise fashion (Figure 7) with disease progression between all disease stage groups (presymptomatic vs. emerging: *p* < 0.001; presymptomatic vs. established: *p* < 0.0001; emerging vs. established: *p* < 0.001). It was also determined that the mitochondria grades for asymmetric synapses were higher than the symmetric synapses at the emerging (*p* < 0.01) and established (*p* < 0.001) disease stages.

### 3.5 Postsynaptic Targeting

**FIGURE 8:**
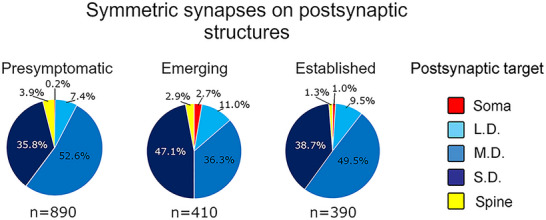
Most presynaptic terminals that formed symmetric synapses did so with small and medium‐sized dendrites. Pie charts showing the distribution of presynaptic terminals forming symmetric synapses onto postsynaptic targets across presymptomatic, emerging, and established disease ICc tissue. The proportion of symmetric synapses onto medium dendrites decreased from presymptomatic to emerging. From emerging to established smaller dendrites were proportionally less likely to be targeted. Presymptomatic—2 months; emerging—8 months; established—15–19 months. Each age group had four cases. LD, large dendrite; MD, medium dendrite; SD, small dendrite.

**FIGURE 9:**
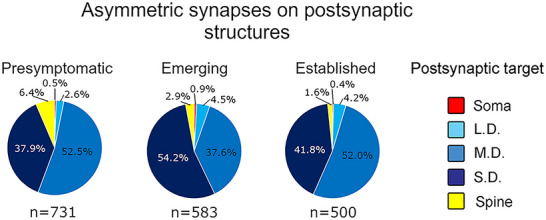
Most presynaptic terminals that formed asymmetric synapses did so with small and medium‐sized dendrites. Pie charts showing the distribution of presynaptic terminals forming asymmetric synapses onto postsynaptic targets across presymptomatic, emerging, and established disease ICc tissue. The proportion of asymmetric synapses onto medium dendrites decreased from presymptomatic to emerging. From emerging to established, smaller dendrites were proportionally less likely to be targeted. Presymptomatic—2 months; emerging—8 months; established—15–19 months. Each age group had four cases. LD, large dendrite; MD, medium dendrite; SD, small dendrite.

For each synapse type, we recorded the postsynaptic target which included somas, dendrites, Sps, and boutons (Figures 8 and 9). Observations of symmetric and asymmetric synapses targeting boutons and unmyelinated axons were uncommon and not represented.

#### 3.5.1 Postsynaptic Targets of Symmetric Synapses

Regardless of disease stage, the most common postsynaptic targets of symmetric synapses were medium and SDs (Figure 8). Collectively, Sps and somas only accounted for 2.3%–5.6% of the postsynaptic targets in any disease stage (Figure 8). Qualitatively, the greatest loss of symmetric synapses between the presymptomatic and emerging disease stages appeared to be those that targeted medium size dendrites (468 presymptomatic synapses vs. 149 emerging synapses; Figure 8). However, the continued synaptic loss from the emerging disease stage to the established disease stage appeared to be driven by a loss of symmetric synapses targeting SDs (Figure 8). Proportionally, the number of terminals forming symmetric synapses on LDs increased from the presymptomatic disease stage (Figure 8).

#### 3.5.2 Postsynaptic Targets of Asymmetric Synapses

Similar to the results reported for symmetric synapses, medium and SDs were qualitatively the most common target regardless of disease stage (Figure  9). Proportionally, LDs increased slightly from presymptomatic to the establish disease stage. Spines were qualitatively a more common postsynaptic target for asymmetric synapses than symmetric synapses, especially during the presymptomatic disease stage (compare Figures 8 and 9).

## 4 Discussion

The current study demonstrates several novel distinctions in 3xTG mouse IC ultrastructure associated with the transition from prepathological to symptomatic disease ages. Overall, these data show that 3xTG mice experienced significant loss of both symmetric and asymmetric synapses in IC with progressing disease stage—however, the temporal pattern of these losses differed by synapse type. At presymptomatic ages, symmetric synapse density exceeded that of asymmetric synapses but by emerging and established disease stages, this pattern was reversed with asymmetric synapses becoming the dominant form in IC. This appeared to result from a substantial reduction in symmetric synapse density in these two disease progression age groups. Although the total loss of asymmetric synapses in 3xTG mice at the established disease stage is very similar to previous reports of loss (∼30%–35%) in the context of normal aging (as measured in old age groups of non‐disease rodent models), the significant loss of symmetric synapses in the 3xTG model was (1) substantially more robust (∼54%) than losses reported in a normal aging model and (2) occurred at an earlier age (Helfert et al. 1999; Wawrzyniak et al. 2025; unpublished observations). This robust early loss of symmetric/inhibitory synapses may create a hyperexcitable environment within the ICc in 3xTG mice. Additional findings in the 3xTG model showed that the (1) average terminal areas forming symmetric and asymmetric synapses significantly increased with age, (2) mitochondrial ultrastructure became poorer with disease progression, and (3) both synaptic types most commonly targeted small‐ and medium‐caliber dendrites at each disease stage. Each of these findings is consistent to what has been reported in the IC during normal aging (Helfert et al. 1999; Wawrzyniak et al. 2025; unpublished observations). Collectively, 3xTG mouse synaptic ultrastructure undergoes age‐related changes reminiscent to normal aging; however, loss of inhibitory synapses is more robust, and the loss of both synaptic types reaches significance in middle age.

### 4.1 Technical Considerations

3xTG mice are a well‐characterized, widely used model of hereditary AD that is considered to have good clinical generalizability (Grimaldi et al. 2018; Belfiore et al. 2019; Oddo et al. 2003; Billings et al. 2005; Frame et al. 2022). Although our 3xTG mice are bred on a hybrid B6129SJ background, and not the C5BL6j background that is well‐known to result in early hearing loss, a caveat of the current study is that we do not have ABR or DPOAE data from the cases in the current study. The 129 inbred strains develop mild ARHL before 13 weeks of age and severe hearing impairments at 40 weeks (Zheng et al. 1999). Given that our emerging and established disease stages are 7/8 months and 15+ months, respectively, it quite reasonable to hypothesize that the 3xTG mice in the current study had some degree of ARHL. It has also been demonstrated that 3xTG mice and their WT controls (B612SJ hybrid) both develop noise‐induced hearing loss; however, in the 3xTG mice there was a reduction in the density of dendritic Sps of auditory cortical cells that did not occur in the B612SJ controls (Paciello et al. 2021). Thus, we acknowledge that the auditory system in our 3xTG mice likely does respond to insult in the same manner as background strain. Thus, these caveats limit the interpretation of our findings being a product of hearing loss or AD. Hopefully future studies will establish whether there is causality between these lost synapses, hearing loss, and AD.

It has been established across multiple models that the vast majority of symmetric synapses in the IC are GABAergic (Shneiderman and Oliver 1989; Helfert et al. 1999; Bartlett et al. 2000; Nakamoto et al. 2013, 2014; Mafi et al. 2022; Wawrzyniak et al. 2025). As GABAergic synapses in the IC are downregulated with age, we generalize that the downregulated symmetric synapses we report in the current study are predominately GABAergic (Helfert et al. 1999; Mafi et al. 2022; Mellott et al. 2024; Wawrzyniak et al. 2025). However, it is possible that a smaller proportion of the symmetric synapses downregulated in the current study represent other neurotransmitters (e.g., glycine, acetylcholine; Liu et al. 1999; Wenstrup and Leroy 2001; Noftz et al. 2024). Thus, we interpret our results to indicate that there is an early robust loss of GABAergic neurotransmission in the 3xTG IC; however, other inhibitory neurotransmitter systems may be represented by these downregulated symmetric synapses.

In the current study, we extracted tissue blocks from the mid‐dorsolateral–ventromedial axis of the ICc. Thus, given that the ICc axis is tonotopically organized, the tissue blocks in this study are likely to better correspond with middle frequencies than high and low frequencies. We chose this approach to allow for better comparison between studies as previous studies of the aging IC ultrastructure also examined regions of tissue that represented the middle frequency regions of the lemniscal input to the IC (Helfert et al. 1999; Mafi et al. 2022; Wawrzyniak et al. 2025). However, unlike our recent study that examined across a greater extent of the dorsolateral–ventromedial axis (Wawrzyniak et al. 2025), the current study is limited as it cannot make statements between higher and lower frequency regions. We may examine these higher and lower regions in subsequent studies, but three factors influenced us to keep the current study more manageable: (1) the time to process and analyze an additional tissue block for each animal is rather substantial, and (2) the mouse IC is small and extracting three tissue blocks that are comfortably within the ICc is rather challenging.

### 4.2 Comparisons to Previous Studies

Understanding the anatomy of the IC in the 3xTG mouse model of AD is critical as (1) it is well established that the downregulation of inhibition in the “normal aging” IC contributes to central gain and subsequent hearing impairments in older populations, (2) studies in the 5xFAD mouse model of AD indicate that central gain may be an early biomarker for AD diagnosis and that AD pathologies occur in the IC, and (3) hearing loss is considered to be a modifiable risk factor for AD (Uhlmann, Teri, et al. 1989; Uhlmann, Larson, et al. 1989; Caspary et al. 2008; Lin et al. 2011; Lin and Albert 2014; Syka 2020; Mukadam et al. 2020; Nadhimi and Llano 2020; Na et al. 2023; Yu et al. 2025). Thus, the impetus behind the current study was to determine if the IC ultrastructure in the 3xTG mouse (a model that develops AD pathologies) ages in a similar manner to what has been documented in the FBN rat (a model routinely used for “normal” aging studies and ARHL; Helfert et al. 1999; Na et al. 2020; Mafi et al. 2022; Wawrzyniak et al. 2025).

One of the more consistent and striking findings between the current study in the 3xTG mouse and previous reports on IC ultrastructure from a normal aging model (FBN rat) is that the density of symmetric/inhibitory synapses and asymmetric/excitatory is largely the same in each respective species at young age (Helfert et al. 1999; Mafi et al. 2022; Wawrzyniak et al. 2025; unpublished data). In each of these previous studies that examined the rat IC, asymmetric synapses accounted for ∼52%–55% of the total synaptic density at the youngest age group, whereas, in the current study, symmetric synapses accounted for 54.9% of the total population during the presymptomatic disease stage (Helfert et al. 1999; Mafi et al. 2022; Wawrzyniak et al. 2025; unpublished data). One of the main findings in Helfert et al. (1999), which our recent study on GABAergic synapses and our unpublished data on asymmetric synapses agree with, is that in the middle frequency region of the ICc, inhibitory and excitatory synapses are lost with age in a balanced manner such that reduction of density for both synaptic types is ∼30%–35% in the oldest age groups (Wawrzyniak et al. 2025). In the current study, symmetric and asymmetric synapses are also downregulated in our AD model, but the significant loss occurred well before the established disease stage group. Collectively, we interpret the data from the current study to imply that while the degree of excitation in the 3xTG mouse ICc is lost in a similar manner to normal aging, the age‐related loss of inhibition is more dramatic and occurs at a much early point in life. As described above, it is quite possible these 3xTG mice have hearing loss. Given that we found a much earlier and robust lost than in the FBN rat, perhaps the downregulated inhibitory synapses in the emerging disease stage are driven by a combination of heading loss and AD pathologies. Interestingly, our previous work in the FBN rat did not show a significant loss of GABAergic synapses in the lower frequency region of the ICc (Wawrzyniak et al. 2025). If this holds true in the 3xTG mouse, future studies will be required to examine the tonotopic axis and to better understand the hearing profile of this model during these various disease stages.

In presymptomatic age 3xTG mice, we found the average size of the presynaptic terminals for symmetric synapses to be 0.63 µm^2^, and 0.5 µm^2^ for asymmetric synapses. Previous studies in the rat ICc measured young presynaptic terminals to range from 0.64 to 0.74 µm^2^ (Mafi et al. 2022; Wawrzyniak et al. 2025). Furthermore, GABAergic and excitatory terminals in the guinea pig ICc were slightly larger on average than in rat (Nakamoto et al. 2014). Although there is plenty of overlap in size across species, it appears that the average size of a presynaptic terminal in the ICc may be proportionally related to the size of the animal. In the context of aging, the current study demonstrated a significant increase in the average size of both synaptic types, but not until the established disease stage. Wawrzyniak et al. (2025) also demonstrated this significant finding regarding as the lost GABAergic terminals often reflected those of smaller areas. As the range of presynaptic terminal sizes did not significantly differ between disease stages in the current study, we interpret the data such that presynaptic terminals of a smaller area are preferentially lost in the aging 3xTG mouse. Perhaps the increase in average terminal size in the current study is reflecting a combination of accelerated loss due to disease progression and a normal mechanism of aging that is not related to AD. As the size of the presynaptic terminal may reflect the origin of input, it will be important for future studies in the 3xTG IC to determine if the lost presynaptic input is from specific ascending, commissural, and/or descending sources.

Another shared result between the current study and our previous investigations is the decline of mitochondrial ultrastructure. Previous studies have demonstrated poorer mitochondrial ultrastructure, in particular compromised cristae, as a sign of disrupted mitophagy (Coughlin et al. 2015; Smith et al. 2016; Youn et al. 2020). We also found that in the current study and our previous studies that mitochondrial ultrastructure becomes poorer with age in the IC (Mafi et al. 2022; Wawrzyniak et al. 2025; Smallridge et al. 2026). Although significance was not reached, the average number of mitochondria in the presynaptic terminals persisted and even trended upward, despite the robust loss of synapses during the emerging and established disease stage. This finding reflected that there were far fewer recorded presynaptic terminals with no mitochondria present as disease progression occurred. Given the prevalence of mitochondria in presynaptic IC terminals, these findings suggest that aging, and the resultant reactive oxygen species, may have substantial consequences on IC metabolism and neurotransmitter release.

Broadly, the results of the current study were similar to previous aging IC studies in that the vast majority of both synaptic types had active zones with smaller and medium‐sized caliber dendrites. It should be noted that the dendritic size criteria are adopted from Helfert et al. (1999), which is in rat along with our previous work. Regardless, synapses in the 3xTG mouse preferentially targeted smaller to medium‐sized caliber dendrites. It was not surprising that we recorded few axosomatic synapses in the current study as ultrastructural studies of the IC in several mammalian models demonstrate that these synaptic inputs are rarer (Ribak and Roberts 1986; Paloff et al. 1992; Kazee et al. 1995; Helfert et al. 1999; Paloff et al. 2004; Nakamoto et al. 2014; Mafi et al. 2022; Wawrzyniak et al. 2025). Studies also demonstrate that axosomatic inputs are more commonly symmetric and/or target larger cells (Oliver and Morest 1984; Ribak and Roberts 1986; Paloff et al. 1992; Nakamoto et al. 2014; Mafi et al. 2022; Wawrzyniak et al. 2025). Interestingly, a subset of GABAergic somas (that are often larger in nature) in the IC are surrounded by a dense network of glutamatergic inputs (Ito et al. 2009; Beebe et al. 2018, 2016). We did not encounter any examples of numerous asymmetric axosomatic synapses, let alone on the same soma, in the current study. The lack of this observation could simply be the nature of EM sampling and the rarity of these GABAergic cells with dense excitatory somatic input. Future studies of the aging and pathological IC may immunolabel for vGlut2 to more readily identify this subset of GABAergic cells.

There were two key differences between what we found in the current study and what has been reported in other species. First, it was demonstrated that in the aging IC that receives dense lemniscal input, there is a synaptic rearrangement such that even though there are less GABAergic synapses, there is a greater number of GABAergic synapses on larger caliber dendrites (Helfert et al. 1999; Mafi et al. 2022; Wawrzyniak et al. 2025). It has yet to be determined what the functional advantage/consequence is of this arrangement in the aging IC. In the current study, this rearrangement did not occur as larger caliber dendrites were not more commonly targeted by symmetric synapses as disease progression occurred. Instead, there appears to be a two‐stage loss of symmetric and asymmetric synapses that target small and MDs as disease progression occurs such synapse loss on MDs during the emerging disease stage is followed by synaptic loss on smaller caliber dendrite during when the disease is established. Although there is a more dramatic loss of symmetric synapses as disease progression occurs, it appears that both synapse types are downregulated in a similar and balanced manner when it comes to the proportion each postsynaptic structure comprises. It would be of value for future studies to determine if extracellular amyloid plaques have a greater likelihood of preventing inhibitory neurotransmission on larger/proximal dendrites.

Second, we have found that active zone lengths trend upward or are significantly larger in our previous studies examining regions of the IC that receive lemniscal inputs (Mafi et al. 2022; Wawrzyniak et al. 2025). As with the above changes to synaptic arrangement, it is unclear the functional changes that result from these morphological changes in the aging IC. In cortex, the lengthening of active zones is viewed as a compensatory mechanism as it occurs with declines in synaptic input (Levine et al. 1988). In our previous studies, the increase to active zone length is curious as the number vesicles within any of the pools or at the active zone does not increase (Mafi et al. 2022; Wawrzyniak et al. 2025). The simple question is then why have a longer active zone and membrane area to release neurotransmitter if there is not an increase of vesicles. Regardless, in the current study active zone lengths did not increase; in fact, if anything, they were shorter in the later disease stage groups.

### 4.3 Functional Implications

It is well documented that the aging IC undergoes significant age‐related plastic changes, some of which are specific to GABAergic neurotransmission ranging from protein expression, GABA_A_ receptor subunit composition, synaptic rearrangement, and synaptic loss (Caspary et al. 2008; Pal et al. 2019; Skya 2020; Ono and Ito 2024). The earlier changes and loss of GABAergic neurotransmission are considered a compensatory mechanism that likely underlies a homeostatic response that allows the IC/brainstem maintain its excitability even though excitation is reduced from the degraded cochlea (Richardson et al. 2012; Caspary et al. 2008; Caspary and Llano 2018). However, these compensatory mechanisms underlie the maladaptive changes that occur later in life as the loss of GABAergic tone is too severe and the imbalance between inhibition and excitation results in central gain that likely contributes to a variety of hearing deficits such as poorer temporal processing/speech processing and perception, hyperacusis, presbycusis, and tinnitus (Willott et al. 1988a, 1988b; Palombi and Caspary 1996; Frisina 2001; Brozoski et al. 2002; Walton et al. 2002; Norena 2011; Parthasarathy and Bartlett 2011; Rabang et al. 2012; Auerbach et al. 2019, 2014; Parthasarathy et al. 2014; Parthasarathy, Bartlett, et al. 2019; Parthasarathy, Herrmann, et al. 2019; Parthasarathy et al. 2020; Caspary and Llano 2018; Ono and Ito 2024). It is beyond the scope of the current study to discuss all the nuances and findings across various models on how aging and hearing loss impact IC function; however, an excellent review on these topics was recently conducted by Ono and Ito (2024).

A recent study by Na et al. (2023) suggests that central gain may act as an early biomarker to diagnose AD in a mouse model (5xFAD) of AD. Specifically, the study found increases to central gain/central excitability before altered auditory brainstem responses and hearing loss, suggesting lesions to the central auditory system preceding peripheral damage (Na et al. 2023). Additionally, amyloid beta plaque deposits in the IC were correlated with the occurrence of central gain (Na et al. 2023). These data support the hypothesis that amyloid plaques in the central auditory system likely disrupt inhibition in the IC, allowing the increase in central gain to be utilized as a biomarker for AD progression and diagnosis. This hypothesis would be in line with previous studies demonstrating that AD symptoms result in inhibitory deficits elsewhere in the brain (Palop et al. 2007; Carvajal and Inestrosa 2011; Verret et al. 2012; Hampel et al. 2018; Xu et al. 2020; Lauterborn et al. 2021). Furthermore, poor inhibitory processing has been proposed as a link between AD and dysfunctional central auditory processing (Pérez‐González et al. 2022).

Numerous studies have demonstrated correlative links between hearing loss and AD; however, the precise mechanisms explaining these associations and why heterogenous population are often afflicted with both neurodegenerative conditions remain elusive (Lin et al. 2011; Lin 2013; Heywood et al. 2017; Ford et al. 2018; Griffiths et al. 2020; Llano et al. 2021; Nadhimi and Llano 2021). The most striking data from the current study was the significant (50%) loss of symmetric synapses in the emerging disease stage. We used female 3xTG mice which have a mean lifespan of 520 ± 200 days (Kane et al. 2018). Given that our emerging disease stage 3xTG mice are 8 months old (∼240 days) and presumably have amyloid plaques with early cognitive deficits (Frame et al. 2023), our data suggest that inhibition in the IC is robustly altered early in the model's lifespan that may parallel the onset of the AD pathologies. Perhaps the GABAergic loss during normal aging that allows for the aforementioned compensation is shortened or does not occur at all in the 3xTG mouse. A logical question to ask is what neurotransmitters are released from these lost symmetric synapses.

Symmetric synapses in the IC are best known to release GABA or glycine (Ribak and Roberts 1986; Roberts and Ribak, 1987; Schneiderman et al., 1989; Paloff and Usunoff 1992; Helfert et al. 1999; Liu et al. 1999; Oliver et al. 2005; Nakamoto et al. 2013, 2014; Mafi et al. 2022; Williams and Ryugo 2024; Wawrzyniak et al. 2025). Disruptions to GABAergic neurotransmission could affect the numerous functions (e.g., temporal processing and sound localization). Glycinergic inputs also have a broad functional role in the IC, but perhaps the best understood roles for glycine in the IC are linked to binaural processing and spectrotemporal integrations (Wenstrup and Leroy 2001; Yavuzoglu et al. 2011; Williams and Ryugo 2024). Additionally, there has been a series of recent experiments characterizing neuropeptide Y (NPY) in the IC (Ni et al. 2015; Silveira et al. 2020, 2023, 2024; Anair et al. 2022; Almassri et al. 2025). Specifically, NPY is expressed in roughly one‐third of IC GABAergic stellate neurons and regulates recurrent excitation (Silveira et al. 2023; Almassri et al. 2025a,b). Whether inhibition may be driven by other peptides/neurotransmitters in the IC is not well known. Given the IC circuits are inhibited by at least three inhibitory neurotransmitters, from over a dozen sources (González‐Hernández et al. 1996; Ono and Ito 2024), it is critical to determine the sources and types of inhibition losing synapses so early during the onset of AD pathologies.

## Author Contributions

Sean R. Hergenrother, Nick J. Tokar, and Miljan Terzic generated data. Jeffrey G. Mellott, Dakota Z. Smallridge, Lena M. Dellaria, and Madeline Guy quantified data. Jesse W. Young performed data analysis. Jeffrey G. Mellott designed the experiments. Jeffrey G. Mellott and Christine M. Dengler‐Crish wrote the article.

## Funding

This study was supported by the NIH/NIDCD grant R01 DC017708 and NIH/NIA grant R03 AG087423. Supported by the American Academy of Neurology Medical Student Research Program.

## Ethics Statement

All applicable international, national, and/or institutional guidelines for the care and use of animals were followed.

## Conflicts of Interest

The authors declare no conflicts of interest.

## Data Availability

The data that support the findings of this study are available from the corresponding author upon reasonable request.

## References

[cne70120-bib-0926] Almassri, L. S. , K. M. Crane , S. R. Hergenrother , et al. 2025a. “Neuropeptide Y mRNA Expression in the Aging Inferior Colliculus of fischer brown norway Rats.” Frontiers in Aging Neuroscience 17: 1626021. 10.3389/fnagi.2025.1626021. 40771198 PMC12326481

[cne70120-bib-0927] Almassri, L. S. , J. C. Harris , K. M. Crane , et al. 2025b. “Ultrastructural Characterization of Neuropeptide Y Synapses in the central Inferior Colliculus of the Fischer Brown Norway Rat.” Neuropeptides 114: 102566. 10.1016/j.npep.2025.102566. 41106237 PMC13271813

[cne70120-bib-0001] Anair, J. D. , M. A. Silveira , P. Mirjalili , N. L. Beebe , B. R. Schofield , and M. T. Roberts . 2022. “Inhibitory NPY Neurons Provide a Large and Heterotopic Commissural Projection in the Inferior Colliculus.” Front Neural Circuits 16: 871924. 10.3389/fncir.2022.871924. 35693026 PMC9178209

[cne70120-bib-0002] Auerbach, B. D. , K. Radziwon , and R. Salvi . 2019. “Testing the Central Gain Model: Loudness Growth Correlates With Central Auditory Gain Enhancement in a Rodent Model of Hyperacusis.” Neuroscience 407: 93–107. 30292765 10.1016/j.neuroscience.2018.09.036 PMC8792806

[cne70120-bib-0003] Auerbach, B. D. , P. V. Rodrigues , and R. J. Salvi . 2014. “Central Gain Control in Tinnitus and Hyperacusis.” Frontiers in Neurology 5: 206. 25386157 10.3389/fneur.2014.00206 PMC4208401

[cne70120-bib-0004] Bartlett, E. L. , J. M. Stark , R. W. Guillery , and P. H. Smith . 2000. “Comparison of the Fine Structure of Cortical and Collicular Terminals in the Rat Medial Geniculate Body.” Neuroscience 100, no. 4: 811–828. 10.1016/s0306-4522(00)00340-7. 11036215

[cne70120-bib-0005] Bates, D. , M. Mächler , B. Bolker , and S. Walker . 2015. “Fitting Linear Mixed‐Effects Models Using lme4.” Journal of Statistical Software 67: 1–48.

[cne70120-bib-0006] Beebe, N. L. , J. G. Mellott , and B. R. Schofield . 2018. “Inhibitory Projections From the Inferior Colliculus to the Medial Geniculate Body Originate From Four Subtypes of GABAergic Cells.” eNeuro 5, no. 5: ENEURO.0406–0418.2018. 10.1523/ENEURO.0406-18.2018. 30456294 PMC6240760

[cne70120-bib-0007] Beebe, N. L. , J. W. Young , J. G. Mellott , and B. R. Schofield . 2016. “Extracellular Molecular Markers and Soma Size of Inhibitory Neurons: Evidence for Four Subtypes of GABAergic Cells in the Inferior Colliculus.” Journal of Neuroscience 36, no. 14: 3988–3999. 10.1523/JNEUROSCI.0217-16.2016. 27053206 PMC4821910

[cne70120-bib-0914] Belfiore, R. , A. Rodin , E. Ferreira , et al. 2019. “Temporal and Regional Progression of Alzheimer's Disease‐Like Pathology in 3xTg‐AD Mice.” Aging Cell 18, no. 1: e12873. 10.1111/acel.12873. 30488653 PMC6351836

[cne70120-bib-0008] Benjamini, Y. , and Y. Hochberg . 1995. “Controlling the False Discovery Rate: A Practical and Powerful Approach to Multiple Testing.” Journal of the Royal Statistical Society Series B 57: 289–300.

[cne70120-bib-0915] Billings, L. M. , S. Oddo , K. N. Green , J. L. McGaugh , and F. M. LaFerla . 2005. “Intraneuronal Abeta Causes the Onset of Early Alzheimer's Disease‐related Cognitive Deficits in Transgenic Mice.” Neuron 45, no. 5: 675–688. 10.1016/j.neuron.2005.01.040. 15748844

[cne70120-bib-0009] Brozoski, T. J. , C. A. Bauer , and D. M. Caspary . 2002. “Elevated Fusiform Cell Activity in the Dorsal Cochlear Nucleus of Chinchillas With Psychophysical Evidence of Tinnitus.” Journal of Neuroscience 22: 2383–2390. 11896177 10.1523/JNEUROSCI.22-06-02383.2002 PMC6758251

[cne70120-bib-0010] Cant, N. B. 2005. “Neuronal Organization in the Inferior colliculus.” In The Inferior Colliculus, edited by J. A. Winer and C. E. Schreiner , 69–114. Springer.

[cne70120-bib-0011] Carvajal, F. J. , and N. C. Inestrosa . 2011. “Interactions of AChE With Aβ Aggregates in Alzheimer's Brain: Therapeutic Relevance of IDN 5706.” Frontiers in Molecular Neuroscience 4: 19. 10.3389/FNMOL.2011.00019. 21949501 PMC3172730

[cne70120-bib-0012] Caspary, D. M. , T. M. Holder , L. F. Hughes , J. C. Milbrandt , R. M. McKernan , and D. K. Naritoku . 1999. “Age‐Related Changes in GABA(A) Receptor Subunit Composition and Function in Rat Auditory System.” Neuroscience 93: 307–312. 10430494 10.1016/s0306-4522(99)00121-9

[cne70120-bib-0013] Caspary, D. M. , L. Ling , J. G. Turner , and L. F. Hughes . 2008. “Inhibitory Neurotransmission, Plasticity and Aging in the Mammalian Central Auditory System.” Journal of Experimental Biology 211: 1781–1791. 18490394 10.1242/jeb.013581 PMC2409121

[cne70120-bib-0014] Caspary, D. M. , and D. A. Llano . 2018. “Aging Process in the Subcortical Auditory System.” In The Oxford Handbook of the Auditory Brainstem, edited by K. Kandler , 1–45. Oxford Press. 10.1093/oxfordhb/9780190849061.013.16.

[cne70120-bib-0902] Chandra, A. , B. A. Levett , S. Waters , et al. 2025. “Evaluating the Link Between Hearing Loss and Alzheimer's Disease Neuropathology: a Systematic Review and Meta‐analysis.” Neurobiology of Aging 154: 92–102. 10.1016/j.neurobiolaging.2025.07.003. 40618486

[cne70120-bib-0015] Christensen, R. H. 2023. Ordinal—Regression Models for Ordinal Data . R Package Version 2023.12‐4.1. CRAN.

[cne70120-bib-0016] Coughlin, L. , R. S. Morrison , P. J. Horner , and D. M. Inman . 2015. “Mitochondrial Morphology Differences and Mitophagy Deficit in Murine Glaucomatous Optic Nerve.” Investigative Ophthalmology & Visual Science 56, no. 3: 1437–1446. 10.1167/iovs.14-16126. 25655803 PMC4347310

[cne70120-bib-0901] Deal, J. A. , J. Betz , K. Yaffe , et al. 2017. “Hearing Impairment and Incident Dementia and Cognitive Decline in Older Adults: the Health ABC Study.” The Journals of Gerontology. Series A, Biological Sciences and Medical Sciences 72, no. 5: 703–709. 10.1093/gerona/glw069. PMC5964742 27071780

[cne70120-bib-0017] Ford, A. H. , G. J. Hankey , B. B. Yeap , J. Golledge , L. Flicker , and O. P. Almeida . 2018. “Hearing Loss and the Risk of Dementia in Later Life.” Maturitas 112: 1–11. 29704910 10.1016/j.maturitas.2018.03.004

[cne70120-bib-0909] Frame, G. , A. Schuller , M. A. Smith , S. D. Crish , and C. M. Dengler‐Crish . 2022. “Alterations in Retinal Signaling across Age and Sex in 3xTg Alzheimer's Disease Mice.” Journal of Alzheimer's Disease: JAD 88, no. 2: 471–492. 10.3233/JAD-220016. 35599482 PMC9398084

[cne70120-bib-0018] Frisina, R. D. 2001. “Subcortical Neural Coding Mechanisms for Auditory Temporal Processing.” Hearing Research 158, no. 1–2: 1–27. 10.1016/s0378-5955(01)00296-9. 11506933

[cne70120-bib-0905] Gates, G. A. , A. Beiser , T. S. Rees , R. B. D'Agostino , and P. A. Wolf . 2002. “Central Auditory Dysfunction May Precede the Onset of Clinical Dementia in People With Probable Alzheimer's Disease.” Journal of the American Geriatrics Society 50, no. 3: 482–488. 10.1046/j.1532-5415.2002.50114.x. 11943044

[cne70120-bib-0019] Gauthier, S. , P. Rosa‐Neto , and J. A. Morais . 2021. World Alzheimer Report: Journey Through the Diagnosis of Dementia. Alzheimer's Disease International. https://www.alzint.org/resource/world‐alzheimer‐report‐2021.

[cne70120-bib-0020] Goman, A. M. , and F. R. Lin . 2016. “Prevalence of Hearing Loss by Severity in the United States.” American Journal of Public Health 106, no. 10: 1820–1822. 10.2105/AJPH.2016.303299. 27552261 PMC5024365

[cne70120-bib-0021] González‐Hernández, T. , B. Mantolán‐Sarmiento , B. González‐González , and H. Pérez‐González . 1996. “Sources of GABAergic Input to the Inferior Colliculus of the Rat.” Journal of Comparative Neurology 372, no. 2: 309–326. 10.1002/(SICI)1096-9861(19960819)372:2<309::AID-CNE11>3.0.CO;2-E. 8863133

[cne70120-bib-0022] Griffiths, T. D. , M. Lad , S. Kumar , et al. 2020. “How Can Hearing Loss Cause Dementia?” Neuron 108, no. 3: 401–412. 10.1016/j.neuron.2020.08.003. 32871106 PMC7664986

[cne70120-bib-0913] Grimaldi, A. , C. Brighi , G. Peruzzi , et al. 2018. “Inflammation, Neurodegeneration and Protein Aggregation in the Retina as Ocular Biomarkers for Alzheimer's Disease in the 3xTg‐AD Mouse Model.” Cell Death & Disease 9, no. 6: 685. 10.1038/s41419-018-0740-5. 29880901 PMC5992214

[cne70120-bib-0928] Guo, Z. C. , J. R. McHaney , A. Parthasarathy , K. A. McFarlane , and B. Chandrasekaran . 2025. “Reduced Neural Distinctiveness of Speech Representations in the Middle‐Aged Brain.” Neurobiology of Language (Cambridge, Mass.), 6. 10.1162/nol_a_00169. PMC12327429 40772235

[cne70120-bib-0024] Hampel, H. , M. M. Mesulam , A. C. Cuello , et al. 2018. “Revisiting the Cholinergic Hypothesis in Alzheimer's Disease: Emerging Evidence From Translational and Clinical Research.” Journal of Prevention of Alzheimer's Disease 61: 2–15. doi: 10.14283/JPAD.2018.43. PMC12280792 30569080

[cne70120-bib-0025] Helfer, K. S. , and E. L. Bartlett . 2020. “Listening to All Voices: Interdisciplinary Approaches to Understanding Hearing in Aging.” Aging and Hearing, edited by K. S. Helfer , E. L. Bartlett , A. N. Popper , and R. R. Fay , 1–8. Springer.

[cne70120-bib-0026] Helfert, R. H. , T. J. Sommer , J. Meeks , P. Hofstetter , and L. F. Hughes . 1999. “Age‐Related Synaptic Changes in the Central Nucleus of the Inferior Colliculus of Fischer‐344 Rats.” Journal of Comparative Neurology 406: 285–298. 10102497

[cne70120-bib-0027] Heywood, R. , Q. Gao , M. S. Z. Nyunt , et al. 2017. “Hearing Loss and Risk of Mild Cognitive Impairment and Dementia: Findings From the Singapore Longitudinal Ageing Study.” Dementia and Geriatric Cognitive Disorders 43: 259–268. 28420004 10.1159/000464281

[cne70120-bib-0028] Ito, T. , D. C. Bishop , and D. L Oliver . 2009. “Two Classes of GABAergic Neurons in the Inferior Colliculus.” Journal of Neuroscience 29, no. 44: 13860–13869. 10.1523/JNEUROSCI.3454-09.2009. 19889997 PMC2814801

[cne70120-bib-0029] Ito, T. , and M. S. Malmierca . 2018. “Neurons, Connections, and Microcircuits of the Inferior Colliculus.” In The Mammalian Auditory Pathways. Springer Handbook of Auditory Research, edited by D. Oliver , N. Cant , R. Fay , and A. Popper , 127–167. Springer. 10.1007/978-3-319-71798-2_6.

[cne70120-bib-0923] Kane, A. E. , S. Shin , A. A. Wong , et al. 2018. “Sex Differences in Healthspan Predict Lifespan in the 3xTg‐AD Mouse Model of Alzheimer's Disease.” Frontiers in Aging Neuroscience 10: 172. 10.3389/fnagi.2018.00172. 29946252 PMC6005856

[cne70120-bib-0030] Kazee, A. M. , L. Y. Han , V. P. Spongr , J. P. Walton , R. J. Salvi , and D. G. Flood . 1995. “Synaptic Loss in the Central Nucleus of the Inferior Colliculus Correlates With Sensorineural Hearing Loss in the C57BL/6 Mouse Model of Presbycusis.” Hearing Research 89: 109–120. 10.1016/0378-5955(95)00128-6. 8600115

[cne70120-bib-0031] Kenward, M. G. , and J. H. Roger . 1997. “Small Sample Inference for Fixed Effects From Restricted Maximum Likelihood.” Biometrics 53: 983–997. 9333350

[cne70120-bib-0032] Kuznetsova, A. , P. B. Brockhoff , and R. H. Christensen . 2017. “lmerTest Package: Tests in Linear Mixed Effects Models.” Journal of Statistical Software 82: 1–26.

[cne70120-bib-0033] Lauterborn, J. C. , P. Scaduto , C. D. Cox , et al. 2021. “Increased Excitatory to Inhibitory Synaptic Ratio in Parietal Cortex Samples From Individuals With Alzheimer's Disease.” Nature Communications 12: 1–15. 10.1038/s41467-021-22742-8. PMC8110554 33972518

[cne70120-bib-0912] Lenth, R. V. 2024. “emmeans: Estimated Marginal Means, aka Least‐Squares Means.” In R Package Version 1103. https://CRAN.R‐project.org/package=emmeans.

[cne70120-bib-0034] Levine, M. S. , A. M. Adinolfi , R. S. Fisher , C. D. Hull , D. Guthrie , and N. A. Buchwald . 1988. “Ultrastructural Alterations in Caudate Nucleus in Aged Cats.” Brain Research 440, no. 2: 267–279. 10.1016/0006-8993(88)90995-x. 3359214

[cne70120-bib-0035] Lin, F. R. , and M. Albert . 2014. “Hearing Loss and Dementia—Who Is Listening?” Aging & Mental Health 18, no. 6: 671–673. 10.1080/13607863.2014.915924. 24875093 PMC4075051

[cne70120-bib-0036] Lin, F. R. , L. Ferrucci , E. J. Metter , Y. An , A. B. Zonderman , and S. M. Resnick . 2011. “Hearing Loss and Cognition in the Baltimore Longitudinal Study of Aging.” Neuropsychology 25, no. 6: 763–770. 10.1037/a0024238. 21728425 PMC3193888

[cne70120-bib-0922] Lin, F. R. , K. Yaffe , J. Xia , et al. and Health ABC Study Group . 2013. “Hearing Loss and Cognitive Decline in Older Adults.” JAMA Internal Medicine 173, no. 4: 293–299. 10.1001/jamainternmed.2013.1868. 23337978 PMC3869227

[cne70120-bib-0038] Liu, Y. , H. Sakurai , K. Kurokawa , H. Yamada , and M. Kudo . 1999. “Glycine‐Immunoreactive Synapses in the Central Nucleus of the Inferior Colliculus. An Electron Microscopic Study in the Cat.” Neuroscience Letters 269, no. 3: 183–185. 10.1016/s0304-3940(99)00448-6. 10454162

[cne70120-bib-0039] Livingston, G. , J. Huntley , K. Y. Liu , et al. 2024. “Dementia Prevention, Intervention, and Care: 2024 Report of the Lancet standing Commission.” Lancet 404, no. 10452: 572–628. 10.1016/S0140-6736(24)01296-0. 39096926

[cne70120-bib-0040] Llano, D. A. , S. S. Kwok , and V. Devanarayan , and Alzheimer's Disease Neuroimaging Initative . 2021. “Reported Hearing Loss in Alzheimer's Disease Is Associated With Loss of Brainstem and Cerebellar Volume.” Frontiers in Human Neuroscience 24, no. 15: 739754. 10.3389/fnhum.2021.739754. PMC8498578 34630060

[cne70120-bib-0903] Loughrey, D. G. , M. E. Kelly , G. A. Kelley , S. Brennan , and B. A. Lawlor . 2018. “Association of Age‐Related Hearing Loss with Cognitive Function, Cognitive Impairment, and Dementia: a Systematic Review and Meta‐analysis.” JAMA otolaryngology– head & neck Surgery 144, no. 2: 115–126. 10.1001/jamaoto.2017.2513. 29222544 PMC5824986

[cne70120-bib-0041] Lüdecke, D. , M. S. Ben‐Shachar , I. Patil , P. Waggoner , and D. Makowski . 2021. “Performance: An R Package for Assessment, Comparison and Testing of Statistical Models.” Journal of Open Source Software 6: 3139.

[cne70120-bib-0042] Mafi, A. M. , N. Tokar , M. G. Russ , O. Barat , and J. G. Mellott . 2022. “Age‐Related Ultrastructural Changes in the Lateral Cortex of the Inferior Colliculus.” Neurobiology of Aging 120: 43–59. 10.1016/j.neurobiolaging.2022.08.007. 36116395 PMC10276896

[cne70120-bib-0043] Mastronarde, D. 2003. “SerialEM: A Program for Automated Tilt Series Acquisition on Tecnai Microscopes Using Prediction of Specimen Position.” Microscopy and Microanalysis 9, no. S02: 1182–1183. 10.1017/S1431927603445911.

[cne70120-bib-0044] Mellott, J. G. , M. E. Bickford , and B. R. Schofield . 2014. “Descending Projections From Auditory Cortex to Excitatory and Inhibitory Cells in the Nucleus of the Brachium of the Inferior Colliculus.” Frontiers in Systems Neuroscience 8: 188. 10.3389/fnsys.2014.00188. 25339870 PMC4186273

[cne70120-bib-0045] Mellott, J. G. , S. Duncan , J. Busby , et al. 2024. “Age‐Related Upregulation of Dense Core Vesicles in the Central Inferior Colliculus.” Frontiers in Cellular Neuroscience 18: 1396387. 10.3389/fncel.2024.1396387. 38774486 PMC11107844

[cne70120-bib-0046] Milbrandt, J. C. , C. Hunter , and D. M. Caspary . 1997. “Alterations of GABAA Receptor Subunit mRNA Levels in the Aging Fischer 344 Rat Inferior Colliculus.” Journal of Comparative Neurology 379: 455–465. 9067836 10.1002/(sici)1096-9861(19970317)379:3<455::aid-cne10>3.0.co;2-f

[cne70120-bib-0904] Mukadam, N. , R. Anderson , M. Knapp , et al. 2020. “Effective Interventions for Potentially Modifiable Risk Factors for Late‐onset Dementia: a Costs and Cost‐effectiveness Modelling Study.” The Lancet. Healthy Longevity 1, no. 1: e13–e20. 10.1016/S2666-7568(20)30004-0. 36094185

[cne70120-bib-0047] Na, D. , J. Zhang , H. J. Beaulac , et al. 2023. “Increased Central Auditory Gain in 5xFAD Alzheimer's Disease Mice as an Early Biomarker Candidate for Alzheimer's Disease Diagnosis.” Frontiers in Neuroscience 17: 1106570. 10.3389/fnins.2023.1106570. 37304021 PMC10250613

[cne70120-bib-0048] Nadhimi, Y. , and D. A. Llano . 2021. “Does Hearing Loss Lead to Dementia? A Review of the Literature.” Hearing Research 402: 108038. 10.1016/j.heares.2020.108038. 32814645 PMC9336511

[cne70120-bib-0049] Nakagawa, S. , P. C. Johnson , and H. Schielzeth . 2017. “The Coefficient of Determination R 2 and Intra‐Class Correlation Coefficient From Generalized Linear Mixed‐Effects Models Revisited and Expanded.” Journal of the Royal Society Interface 14: 20170213. 28904005 10.1098/rsif.2017.0213 PMC5636267

[cne70120-bib-0050] Nakamoto, K. T. , J. G. Mellott , J. Killius , M. E. Storey‐Workley , C. S. Sowick , and B. R. Schofield . 2013. “Ultrastructural Examination of the Corticocollicular Pathway in the Guinea Pig: A Study Using Electron Microscopy, Neural Tracers, and GABA Immunocytochemistry.” Frontiers in Neuroanatomy 7: 13. 10.3389/fnana.2013.00013. 23734104 PMC3660666

[cne70120-bib-0051] Nakamoto, K. T. , J. G. Mellott , J. Killius , M. E. Storey‐Workley , C. S. Sowick , and B. R. Schofield . 2014. “Ultrastructural Characterization of GABAergic and Excitatory Synapses in the Inferior Colliculus.” Frontiers in Neuroanatomy 8: 108. 10.3389/fnana.2014.00108. 25400551 PMC4212260

[cne70120-bib-0925] Ni, R. J. , Y. M. Shu , P. H. Luo , et al. 2015. “Immunohistochemical Mapping of Neuropeptide Y in the Tree Shrew Brain.” The Journal of Comparative Neurology 523, no. 3: 495–529. 10.1002/cne.23696. 25327585

[cne70120-bib-0916] Noftz, W. A. , E. E. Echols , N. L. Beebe , J. G. Mellott , and B. R. Schofield . 2024. “Differential Cholinergic Innervation of Lemniscal versus Non‐lemniscal Regions of the Inferior Colliculus.” Journal of Chemical Neuroanatomy 139: 102443. 10.1016/j.jchemneu.2024.102443. 38914378 PMC11827475

[cne70120-bib-0052] Norena, A. J. 2011. “An Integrative Model of Tinnitus Based on a Central Gain Controlling Neural Sensitivity.” Neuroscience & Biobehavioral Reviews 35: 1089–1109. 21094182 10.1016/j.neubiorev.2010.11.003

[cne70120-bib-0910] Oddo, S. , A. Caccamo , M. Kitazawa , B. P. Tseng , and F. M. LaFerla . 2003. “Amyloid Deposition Precedes Tangle Formation in a Triple Transgenic Model of Alzheimer's disease.” Neurobiology of Aging 24, no. 8: 1063–1070. 10.1016/j.neurobiolaging.2003.08.012. 14643377

[cne70120-bib-0053] Oliver, D. L. 2005. “Neuronal Organization in the Inferior Colliculus.” In The Inferior Colliculus, edited by J. A. Winer and C. E. Schreiner , 69–114. Springer.

[cne70120-bib-0054] Oliver, D. L. , and D. K. Morest . 1984. “The Central Nucleus of the Inferior Colliculus in the Cat.” Journal of Comparative Neurology 222, no. 2: 237–264. 10.1002/cne.902220207. 6699209

[cne70120-bib-0055] Ono, M. , and T. Ito . 2024. “Hearing Loss‐Related Altered Neuronal Activity in the Inferior Colliculus.” Hearing Research 449: 109033. 10.1016/j.heares.2024.109033. 38797036

[cne70120-bib-0056] Paciello, F. , M. Rinaudo , V. Longo , et al. 2021. “Auditory Sensory Deprivation Induced by Noise Exposure Exacerbates Cognitive Decline in a Mouse Model of Alzheimer's Disease.” Elife 10: e70908. 10.7554/eLife.70908. 34699347 PMC8547960

[cne70120-bib-0057] Pal, I. , C. R. B. Paltati , C. Kaur , et al. 2019. “Morphological and Neurochemical Changes in GABAergic Neurons of the Aging Human Inferior Colliculus.” Hearing Research 377: 318–329. 10.1016/j.heares.2019.02.005. 30878270

[cne70120-bib-0058] Paloff, A. M. , and K. G. Usunoff . 1992. “The Fine Structure of the Inferior Colliculus in the Cat II. Synaptic Organization.” Journal Fur Hirnforschung 33: 77–106. 1447517

[cne70120-bib-0059] Paloff, A. M. , K. G. Usunoff , P. Yotovski , D. V. Hinova‐Palova , and W. A. Ovtscharoff . 2004. “Parvalbumin‐Like Immunostaining in the Cat Inferior Colliculus. Light and Electron Microscopic Investigation.” Acta Histochemica 106, no. 3: 219–234. 10.1016/j.acthis.2003.11.006. 15186929

[cne70120-bib-0060] Palombi, P. S. , and D. M. Caspary . 1996. “Responses of Young and Aged Fischer 344 Rat Inferior Colliculus Neurons to Binaural Tonal Stimuli.” Hearing Research 100, no. 1–2: 59–67. 10.1016/0378-5955(96)00113-x. 8922980

[cne70120-bib-0919] Paloff, A. M. , K. G. Usunoff , and D. V. Hinova‐Palova . 1992. “Ultrastructure of Golgi‐impregnated and Gold‐toned Neurons in the central Nucleus of the Inferior Colliculus in the Cat.” Journal Fur Hirnforschung 33, no. 4‐5: 361–407. 1282528

[cne70120-bib-0061] Palop, J. J. , J. Chin , E. D. Roberson , et al. 2007. “Aberrant Excitatory Neuronal Activity and Compensatory Remodeling of Inhibitory Hippocampal Circuits in Mouse Models of Alzheimer's Disease.” Neuron 55: 697–711. 10.1016/J.NEURON.2007.07.025/ATTACHMENT/CE669DDE-0DFB-4708-997D-5949953A82B2/MMC1. 17785178 PMC8055171

[cne70120-bib-0062] Parthasarathy, A. , and E. L. Bartlett . 2011. “Age‐Related Auditory Deficits in Temporal Processing in F‐344 Rats.” Neuroscience 192: 619–630. 10.1016/j.neuroscience.2011.06.042. 21723376

[cne70120-bib-0063] Parthasarathy, A. , E. L. Bartlett , and S. G. Kujawa . 2019. “Age‐Related Changes in Neural Coding of Envelope Cues: Peripheral Declines and Central Compensation.” Neuroscience 407: 21–31. 10.1016/j.neuroscience.2018.12.007. 30553793 PMC8600413

[cne70120-bib-0064] Parthasarathy, A. , J. Datta , J. A. L. Torres , C. Hopkins , and E. L. Bartlett . 2014. “Age‐Related Changes in the Relationship Between Auditory Brainstem Responses and Envelope Following Responses.” Journal of the Association for Research in Otolaryngology 15: 649–661. 10.1007/s10162-014-0460-1. 24845405 PMC4141432

[cne70120-bib-0065] Parthasarathy, A. , B. Herrmann , and E. L. Bartlett . 2019. “Aging Alters Envelope Representations of Speech‐Like Sounds in the Inferior Colliculus.” Neurobiology of Aging 73: 30–40. 10.1016/j.neurobiolaging.2018.08.023. 30316050 PMC6251750

[cne70120-bib-0067] Parthasarathy, A. , S. Romero Pinto , R. M. Lewis , W. Goedicke , and D. B. Polley . 2020. “Data‐Driven Segmentation of Audiometric Phenotypes Across a Large Clinical Cohort.” Science Report 10, no. 1: 6704. 10.1038/s41598-020-63515-5. PMC7174357 32317648

[cne70120-bib-0068] Paxinos, G. , and K. B. J. Franklin . 2019. The Mouse Brain in Stereotaxic Coordinates. Academic Press.

[cne70120-bib-0069] Pérez‐González, D. , T. G. Schreiner , D. A. Llano , and M. S. Malmierca . 2022. “Alzheimer's Disease, Hearing Loss, and Deviance Detection.” Frontiers in Neuroscience 16: 879480. 10.3389/fnins.2022.879480. 35720686 PMC9201340

[cne70120-bib-0071] Rabang, C. F. , A. Parthasarathy , Y. Venkataraman , Z. L. Fisher , S. M. Gardner , and E. L. Bartlett . 2012. “A Computational Model of Inferior Colliculus Responses to Amplitude Modulated Sounds in Young and Aged Rats.” Frontiers in Neural Circuits 6: 77. 23129994 10.3389/fncir.2012.00077 PMC3487458

[cne70120-bib-0072] Reynolds, E. S. 1963. “The Use of Lead Citrate at High pH as an Electron‐Opaque Stain in Electron Microscopy.” Journal of Cell Biology 17: 208–212. 13986422 10.1083/jcb.17.1.208 PMC2106263

[cne70120-bib-0073] Ribak, C. E. , and R. C. Roberts . 1986. “The Ultrastructure of the Central Nucleus of the Inferior Colliculus of the Sprague–Dawley Rat.” Journal of Neurocytology 15, no. 4: 421–438. 10.1007/BF01611726. 3746353

[cne70120-bib-0074] Richardson, B. D. , T. J. Brozoski , L. L. Ling , and D. M. Caspary . 2012. “Targeting Inhibitory Neurotransmission in Tinnitus.” Brain Research 1485: 77–87. 22405692 10.1016/j.brainres.2012.02.014 PMC3374875

[cne70120-bib-0075] Roberts, R. C. , and C. E. Ribak . 1987. “An Electron Microscopic Study of GABAergic Neurons and Terminals in the Central Nucleus of the Inferior Colliculus of the Rat.” Journal of Neurocytology 16, no. 3: 333–345. 10.1007/BF01611345. 3302119

[cne70120-bib-0911] R Core Team . 2025. R: a Language and Environment for Statistical Computing. R Foundation for Statistical Computing. https://www.r‐project.org/.

[cne70120-bib-0076] Robinson, L. C. , O. Barat , and J. G. Mellott . 2019. “GABAergic and Glutamatergic Cells in the Inferior Colliculus Dynamically Express the GABAAR γ1subunit During Aging.” Neurobiology of Aging 80: 99–110. 10.1016/j.neurobiolaging.2019.04.007. 31112831

[cne70120-bib-0077] Rockel, A. J. , and E. G. Jones . 1973. “Observations on the Fine Structure of the Central Nucleus of the Inferior Colliculus of the Cat.” Journal of Comparative Neurology 147: 61–92. 4682184 10.1002/cne.901470104

[cne70120-bib-0906] Rüb, U. , K. Stratmann , H. Heinsen , et al. 2016. “The Brainstem Tau Cytoskeletal Pathology of Alzheimer's Disease: a Brief Historical Overview and Description of Its Anatomical Distribution Pattern, Evolutional Features, Pathogenetic and Clinical Relevance.” Current Alzheimer Research 13, no. 10: 1178–1197. 10.2174/1567205013666160606100509. 27264543

[cne70120-bib-0078] Schneider, C. , W Rasband , and K. Eliceiri . 2012. “NIH Image to ImageJ: 25 Years of Image Analysis.” Nature Methods 9: 671–675. 10.1038/nmeth.2089. 22930834 PMC5554542

[cne70120-bib-0079] Schofield, B. R. 2005. “Superior Olivary Complex and Lateral Lemniscal Connections of the Auditory Midbrain.” In The Inferior Colliculus, edited by J. A. Winer and C. E. Schreiner , 132–154. Springer.

[cne70120-bib-0080] Shneiderman, A. , and D. L. Oliver . 1989. “EM Autoradiographic Study of the Projections From the Dorsal Nucleus of the Lateral Lemniscus: A Possible Source of Inhibitory Inputs to the Inferior Colliculus.” Journal of Comparative Neurology 286, no. 1: 28–47. 10.1002/cne.902860103. 2768557

[cne70120-bib-0924] Shneiderman, A. , and D. L. Oliver . 1989. “EM Autoradiographic Study of the Projections From the Dorsal Nucleus of the Lateral Lemniscus: a Possible Source of Inhibitory Inputs to the Inferior Colliculus.” J Comp Neurol. 286, no. 1: 28–47. 10.1002/cne.902860103. 2768557

[cne70120-bib-0081] Silveira, M. A. , J. D. Anair , N. L. Beebe , P. Mirjalili , B. R. Schofield , and M. T. Roberts . 2020. “Neuropeptide Y Expression Defines a Novel Class of GABAergic Projection Neuron in the Inferior Colliculus.” Journal of Neuroscience: The Official Journal of the Society for Neuroscience 40, no. 24: 4685–4699. 32376782 10.1523/JNEUROSCI.0420-20.2020 PMC7294802

[cne70120-bib-0082] Silveira, M. A. , A. C. Drotos , T. M. Pirrone , T. S. Versalle , A. Bock , and M. T. Roberts . 2023. “Neuropeptide Y Signaling Regulates Recurrent Excitation in the Auditory Midbrain.” Journal of Neuroscience: The Official Journal of the Society for Neuroscience 43, no. 45: 7626–7641. 10.1523/JNEUROSCI.0900-23.2023. 37704372 PMC10634549

[cne70120-bib-0083] Silveira, M. A. , Y. N. Herrera , N. L. Beebe , B. R. Schofield , and M. T. Roberts . 2024. “Lineage‐Tracing Reveals an Expanded Population of NPY Neurons in the Inferior Colliculus.” Journal of Neurophysiology 132, no. 2: 573–588. 10.1152/jn.00131.2024. 38988288 PMC11427056

[cne70120-bib-0918] Smallridge, D. Z. , K. Tenney , G. L. Barach , et al. 2026. “Age‐related Ultrastructural Differences in the Dorsal Cortex of the Inferior Colliculus in the fischer Brown Norway Rat.” Neurobiology of Aging 157: 1–16. 10.1016/j.neurobiolaging.2025.09.008. 40997654 PMC12797297

[cne70120-bib-0084] Smith, M. A. , C. Z. Xia , C. M. Dengler‐Crish , et al. 2016. “Persistence of Intact Retinal Ganglion Cell Terminals After Axonal Transport Loss in the DBA/2J Mouse Model of Glaucoma.” Journal of Comparative Neurology 524: 3503–3517. 10.1002/cne.24012. 27072596 PMC5050057

[cne70120-bib-0085] Syka, J. 2020. “Age‐Related Changes in the Auditory Brainstem and Inferior Colliculus.” Aging and Hearing, edited by K. S. Helfer , E. L. Bartlett , A. N. Popper , and R. R. Fay , 67–96. Springer.

[cne70120-bib-0086] Tsui, K. C. , J. Roy , S. C. Chau , et al. 2022. “Distribution and Inter‐Regional Relationship of Amyloid‐Beta Plaque Deposition in a 5xFAD Mouse Model of Alzheimer's Disease.” Frontiers in Aging Neuroscience 14: 964336. 10.3389/fnagi.2022.964336. 35966777 PMC9371463

[cne70120-bib-0087] Uhlmann, R. F. , E. B. Larson , T. S. Rees , T. D. Koepsell , and L. G Duckert . 1989. “Relationship of Hearing Impairment to Dementia and Cognitive Dysfunction in Older Adults.” Jama 261, no. 13: 1916–1919. 2926927

[cne70120-bib-0088] Uhlmann, R. F. , L. Teri , T. S. Rees , K. J. Mozlowski , and E. B Larson . 1989. “Impact of Mild to Moderate Hearing Loss on Mental Status Testing. Comparability of Standard and Written Mini‐Mental State Examinations.” Journal of the American Geriatrics Society 37, no. 3: 223–228. 10.1111/j.1532-5415.1989.tb06811.x. 2918192

[cne70120-bib-0089] Verret, L. , E. O. Mann , G. B. Hang , et al. 2012. “Inhibitory Interneuron Deficit Links Altered Network Activity and Cognitive Dysfunction in Alzheimer Model.” Cells 149: 708–721. 10.1016/J.CELL.2012.02.046. PMC3375906 22541439

[cne70120-bib-0090] Walton, J. P. , H. Simon , and R. D. Frisina . 2002. “Age‐Related Alterations in the Neural Coding of Envelope Periodicities.” Journal of Neurophysiology 88: 565–578. 10.1152/jn.2002.88.2.565. 12163510

[cne70120-bib-0908] Wawrzyniak, A. , J. Busby , A. Dalo , et al. 2025. “Age‐related Differences in GABAergic Synapses Across the central Inferior Colliculus in the Fischer Brown Norway Rat.” Neurobiology of Aging 153: 30–48. 10.1016/j.neurobiolaging.2025.06.002. 40505595 PMC13263886

[cne70120-bib-0091] Wenstrup, J. , and S. A. Leroy . 2001. “Spectral Integration in the Inferior Colliculus: Role of Glycinergic Inhibition in Response Facilitation.” Journal of Neuroscience 21, no. 3: RC124. 10.1523/JNEUROSCI.21-03-j0002.2001. 11157095 PMC6762325

[cne70120-bib-0092] Wickham, H. , M. Averick , J. Bryan , et al. 2019. “Welcome to the Tidyverse.” Journal of Open Source Software 4: 1686.

[cne70120-bib-0093] Williams, I. R. , and D. K. Ryugo . 2024. “Bilateral and Symmetric Glycinergic and Glutamatergic Projections From the LSO to the IC in the CBA/CaH Mouse.” Frontiers in Neural Circuits 18: 1430598. 10.3389/fncir.2024.1430598. 39184455 PMC11341401

[cne70120-bib-0920] Willott, J. F. , K. Parham , and K. P. Hunter . 1988a. “Response Properties of Inferior Colliculus Neurons in Middle‐aged C57BL/6J Mice With Presbycusis.” Hear Res. 37, no. 1: 15–27. 10.1016/0378-5955(88)90074-3. 3225229

[cne70120-bib-0921] Willott, J. F. , K. Parham , and K. P. Hunter . 1988b. “Response Properties of Inferior Colliculus Neurons in Young and Very Old CBA/J Mice.” Hear Res. 37, no. 1: 1–14. 10.1016/0378-5955(88)90073-1. 3225228

[cne70120-bib-0094] Wu, P. Z. , J. T. O'Malley , V. de Gruttola , and M. C. Liberman . 2020. “Age‐Related Hearing Loss Is Dominated by Damage to Inner Ear Sensory Cells, Not the Cellular Battery That Powers Them.” Journal of Neuroscience 40, no. 33: 6357–6366. 10.1523/JNEUROSCI.0937-20.2020. 32690619 PMC7424870

[cne70120-bib-0095] Wu, P. Z. , J. T. O'Malley , and M. C. Liberman . 2023. “Neural Degeneration in Normal‐Aging Human Cochleas: Machine‐Learning Counts and 3D Mapping in Archival Sections.” Journal of the Association for Research in Otolaryngology 24, no. 5: 499–511. 10.1007/s10162-023-00909-y. 37957485 PMC10695900

[cne70120-bib-0096] Xu, Y. , M. Zhao , Y. Han , and H. Zhang . 2020. “GABAergic Inhibitory Interneuron Deficits in Alzheimer's Disease: Implications for Treatment.” Frontiers in Neuroscience 14: 660. 10.3389/FNINS.2020.00660. 32714136 PMC7344222

[cne70120-bib-0097] Yavuzoglu, A. , B. R. Schofield , and J. J. Wenstrup . 2011. “Circuitry Underlying Spectrotemporal Integration in the Auditory Midbrain.” Journal of Neuroscience 31, no. 40: 14424–14435. 10.1523/JNEUROSCI.3529-11.2011. 21976527 PMC3226782

[cne70120-bib-0917] Youn, C. K. , Y. Jun , E. R. Jo , and S. I. Cho . 2020. “Age‐Related Hearing Loss in C57BL/6J Mice Is Associated With Mitophagy Impairment in the Central Auditory System.” International Journal of Molecular Sciences 21, no. 19: 7202. 10.3390/ijms21197202. 33003463 PMC7584026

[cne70120-bib-0098] Yu, R. C. , M. Pavlou , A. G. M. Schilder , et al. 2025. “Early Detection and Management of Hearing Loss to Reduce Dementia Risk in Older Adults With Mild Cognitive Impairment: Findings From the Treating Auditory Impairment and Cognition Trial (TACT).” Age and Ageing 54, no. 1: afaf004. 10.1093/ageing/afaf004. 39835654 PMC11747994

[cne70120-bib-0099] Zheng, M. , J. Yan , W. Hao , et al. 2022. “Worsening Hearing Was Associated With Higher β‐Amyloid and Tau Burden in Age‐Related Hearing Loss.” Scientific Reports 12, no. 1: 10493. 10.1038/s41598-022-14466-6. 35729211 PMC9212197

[cne70120-bib-0100] Zheng, Q. Y. , K. R. Johnson , and L. C. Erway . 1999. “Assessment of Hearing in 80 Inbred Strains of Mice by ABR Threshold Analyses.” Hearing Research 130, no. 1–2: 94–107. 10.1016/s0378-5955(99)00003-9. 10320101 PMC2855304

[cne70120-bib-0101] Zink, M. E. , L. Zhen , J. R. McHaney , et al. 2025. “Increased Listening Effort and Cochlear Neural Degeneration Underlie Speech‐in‐Noise Deficits in Normal‐Hearing Middle‐Aged Adults.” Elife 13: RP102823. 10.7554/eLife.102823. 40694041 PMC12283073

